# Human Gingival Fibroblasts as a Novel Cell Model Describing the Association between Bitter Taste Thresholds and Interleukin-6 Release

**DOI:** 10.1021/acs.jafc.2c06979

**Published:** 2023-03-21

**Authors:** Johanna Tiroch, Andreas Dunkel, Sonja Sterneder, Sofie Zehentner, Maik Behrens, Antonella Di Pizio, Jakob P. Ley, Barbara Lieder, Veronika Somoza

**Affiliations:** † Department of Physiological Chemistry, Faculty of Chemistry, University of Vienna, Vienna 1090, Austria; ‡ Vienna Doctoral School in Chemistry (DoSChem), University of Vienna, Vienna 1090, Austria; § Leibniz Institute for Food Systems Biology at the Technical University of Munich, Freising 85354, Germany; ∥ Symrise AG, Holzminden 37603, Germany; ⊥ Chair for Nutritional Systems Biology, Technical University Munich, Freising 85354, Germany

**Keywords:** human gingival cells (HGF-1), bitter taste threshold, bitter taste receptors, bitter taste modulators, interleukin-6

## Abstract

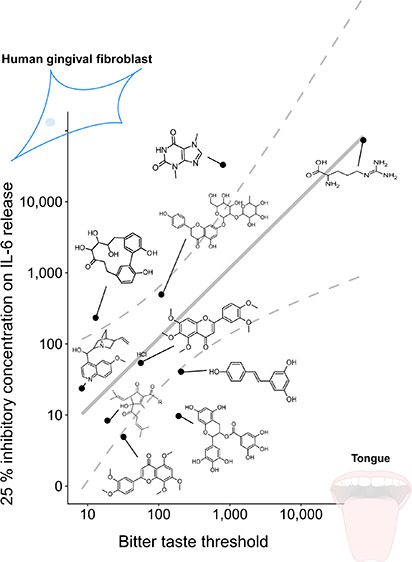

Human gingival fibroblast cells (HGF-1 cells) present an important cell model to investigate the gingiva’s response to inflammatory stimuli such as lipopolysaccharides from *Porphyromonas gingivalis* (*Pg-*LPS). Recently, we demonstrated *trans*-resveratrol to repress the *Pg*-LPS evoked release of the pro-inflammatory cytokine interleukin–6 (IL-6) via involvement of bitter taste sensing receptor TAS2R50 in HGF-1 cells. Since HGF-1 cells express most of the known 25 TAS2Rs, we hypothesized an association between a compound’s bitter taste threshold and its repressing effect on the *Pg*-LPS evoked IL-6 release by HGF-1 cells. To verify our hypothesis, 11 compounds were selected from the chemical bitter space and subjected to the HGF-1 cell assay, spanning a concentration range between 0.1 μM and 50 mM. In the first set of experiments, the specific role of TAS2R50 was excluded by results from structurally diverse TAS2R agonists and antagonists and by means of a molecular docking approach. In the second set of experiments, the HGF-1 cell response was used to establish a linear association between a compound’s effective concentration to repress the *Pg*-LPS evoked IL-6 release by 25% and its bitter taste threshold concentration published in the literature. The Pearson correlation coefficient revealed for this linear association was *R*^2^ = 0.60 (*p* < 0.01), exceeding respective data for the test compounds from a well-established native cell model, the HGT-1 cells, with *R*^2^ = 0.153 (*p* = 0.263). In conclusion, we provide a predictive model for bitter tasting compounds with a potential to act as anti-inflammatory substances.

## Introduction

Fibroblasts are located in connective tissues and represent an important key player in the inflammatory responses to external stimuli.^1^ They are involved in processes of tissue repair via the extra cellular matrix as well as regulating processes during acute and adaptive immunity.^2−4^ Human gingival fibroblasts of the HGF-1 cell line are well established for the identification of anti-inflammatory plant components by reducing the release of the pro-inflammatory proteins tumor necrosis factor α (TNF-α), interleukin-8 (IL-8), and interleukin-6 (IL-6)^5−9^ after exposure to lipopolysaccharides (LPS).^6^ Expression of human bitter taste receptors (TAS2Rs) and their involvement in inflammatory processes in HGF-1 cells has been demonstrated by the following two very recent studies: Zhou et al. demonstrated HGF-1 cells (i) to express TAS2Rs and downstream signaling proteins, and (ii) the activation of TAS2R16 signaling by salicin to inhibit the release of LPS-induced pro-inflammatory cytokine IL-8 by repressing LPS-induced intracellular cAMP elevation and NF-κB p65 nuclear translocation.^10^ In one of our own previous studies, TAS2R50 showed an involvement in the *trans*-resveratrol-mediated IL-6 response of HGF-1 cells to a 6 h treatment with LPS isolated from the Gram-negative bacterium *Porphyromonas gingivalis* (*Pg*-LPS).^9^ In the study presented here, we aimed at elucidating whether the impact on the *Pg*-LPS-induced IL-6 release by HGF-1 cells is associated with the bitter taste threshold of taste active food compounds.

The perception of bitter taste is initiated by agonist–receptor interactions of orally ingested compounds and TAS2Rs, which are expressed in taste buds of the oral cavity.^11,12^ In humans, the 25 TAS2Rs are G protein-coupled receptors (GPCRs).^13^ Analytical methods for the identification of bitter tasting compounds targeting TAS2Rs traditionally comprise sensory procedures,^14^ for which safety data and amounts in the milligram range are required. Sensory studies are limited by the sensory fatigue of panelists, with a relatively low number of compounds that can be tested per day, which makes sensory testing a relatively slow and low throughput method. Sensory methods targeted to identify compounds with a lower recognition threshold might not capture bitter tasting compounds with high threshold concentrations. Therefore, the entire chemical space of bitter tasting compounds might not have been deciphered yet. This presents a major limitation not only for the understanding but also for modulation of bitter tastes of foods and food formulations.

The discovery of TAS2Rs has initiated various *in silico* and *in vitro* approaches for the identification of bitter tasting compounds. *In silico* strategies build on structural similarities between in vitro and sensory-evaluated bitter tastants and their TAS2R molecular interaction properties, as already compiled in several databases, e.g., BitterDB^15^ or FSBI-DB.^16^ One of the most recent works by Bayer et al.^17^ presented a systematic chemoinformatic analysis of the patterns of identified TAS2R agonists,^18,19^ providing a detailed characterization of associations between the chemical properties of bitter tastants in foods and TAS2Rs and a framework for chemoinformatic work on the growing number of food bitter compounds.

Although *in silico* approaches benefit from independence from human sensory panelists and the associated requirement for quantities of safe-to-consume bitter tastants, sensory validation of novel, potentially bitter tasting compounds identified is still required. One of the main reasons for the divergence between *in silico* results and sensory perception is that chemoinformatic strategies mostly integrate data from multiple agonists targeting a given TAS2R, whereas the gustatory perception of bitterness is generated by either activation of individual specific TAS2Rs, e.g., TAS2R7,^20,21^ TAS2R16,^22,23^ TAS2R38,^24,25^ or the complex interplay of the ∼25 TAS2Rs expressed in the human oral cavity.^12^

The implementation of cell-based *in vitro* screenings provides another strategy for the identification of novel tastants and taste modifiers.^26^ Compared to sensory studies, cell-based assays can be performed at high throughput, irrespective of the toxicological safety of compounds, and can help to replace time-consuming human sensory profiling.

With further progress and insights into the taste signaling pathway of mammalian cells, native cell-based assays founded on immortal human cell lines, which endogenously express taste receptor genes from nontaste tissue, have been established. These cell-based assays represent the native transduction signaling pathways and enable the identification of agonists being active on a multi-receptor level, as well as on the native cell context, which may offer an association between the sensory perception and the cellular response to agonist–receptor activation.

For screening approaches, one of the native cell systems established for the identification of bitter tasting and bitter taste modulating compounds is based on the proton secretion by human parietal cells of the HGT-1 cell line, which is linked to the agonist-forced activation of TAS2Rs.^27−29^ Since one of our recent studies demonstrated that TAS2R50 mediates an anti-inflammatory effect on IL-6 release in human gingival fibroblasts of the HGF-1 cell line induced by the bitter tasting *trans*-resveratrol (RSV),^9^ we hypothesized an association of the bitter taste threshold of food constituents and their impact on the *Pg*-LPS-induced IL-6 release by HGF-1 cells. To test this hypothesis, we present a chemoinformatic approach for agonist selection combined with a native cell-based screening. In a first set of experiments, we tested whether repression of the *Pg*-LPS-induced IL-6 release by HGF-1 cells by bitter tasting compounds is specific for compounds targeting TAS2R50. Prior to these experiments, the HGF-1 cells studied were verified for the protein expression of guanine nucleotide-binding protein G(i) subunit alpha-3 (GNAI3) as an important protein for tissue and dentin regeneration in stem cells from the apical papilla (SCAPs)^30^ as well as one subunit of the TAS2R signaling proteins.^31^

**Figure 1:**
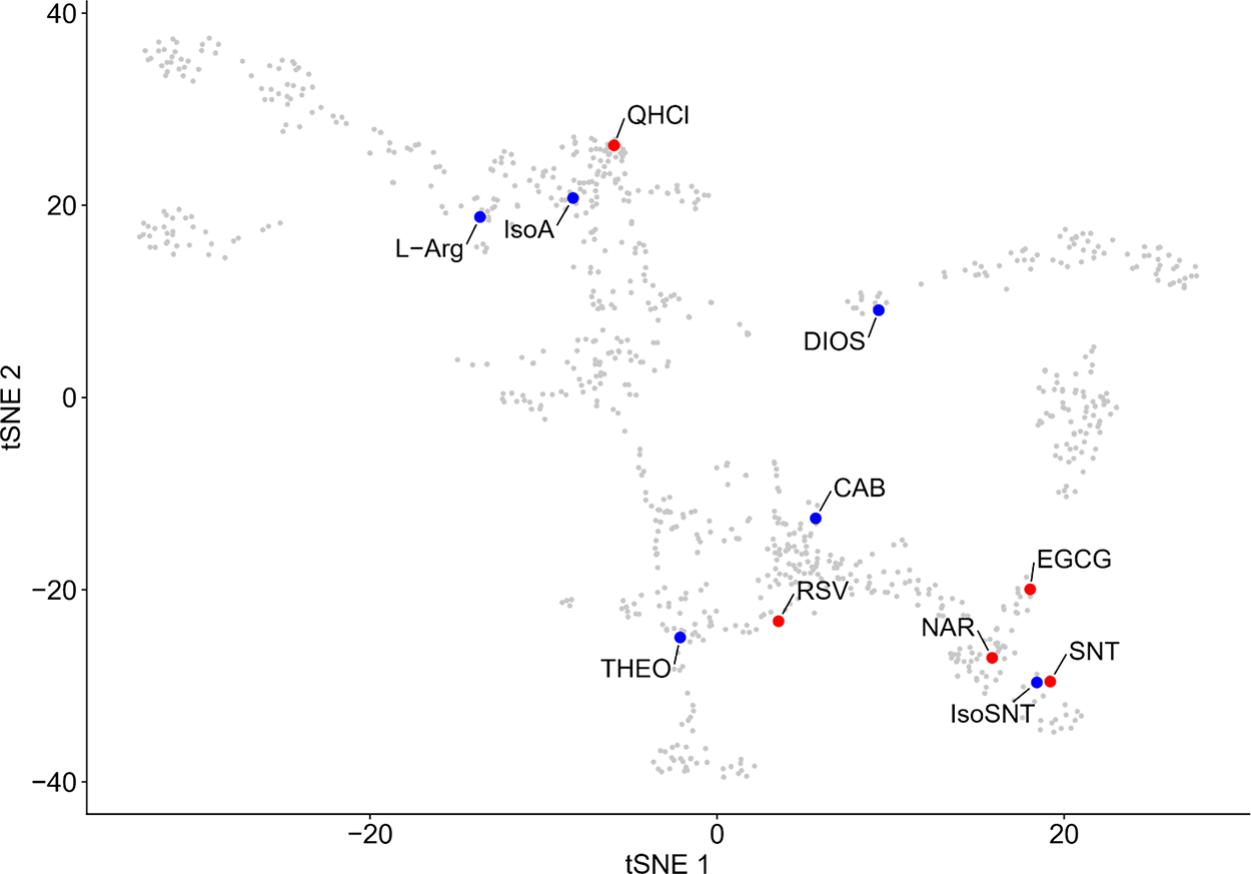
Selected substances (red, first selection; blue, second selection) and their mapped localization in the 2D t-SNE plot of the bitter chemical space^32^ (gray).

Based on our previous study, demonstrating RSV as an effective compound,^9^ four structurally similar (sinensetin, SNT; epigallocatechin gallate, EGCG; naringin, NAR), and one structurally diverging (quinine, QHCl) TAS2R agonist were selected from the chemical bitter space (Figure 1, red circles)^32^ to cover a wider range of TAS2Rs targeted. Next to the impact of these compounds on the *Pg*-LPS-induced IL-6 release in HGF-1 cells, their TAS2R50 binding potential was studied by means of molecular docking simulations. Since these results revealed no specific TAS2R50 involvement, which is in agreement with previous screening results,^18,33^ the HGF-1 cell assay was conducted in an experimental setting of co-incubations, applying the abovementioned TAS2R agonists and the antagonists homoeriodictyol (HED,^34^ targeting TAS2R20/31/43/50^27^) and matairesinol (MAT, demonstrated in this publication to target TAS2R4/43). As these results verified involvement of a wide range of TAS2Rs, we hypothesized the repression of LPS-induced IL-6 release in HGF-1 cells to be associated with the bitter taste quality of a food constituent, namely, its bitter taste threshold for which literature data are available. This hypothesis was tested in a second set of experiments, which included additional substances with higher structural diversity from the chemical bitter space (Figure 1, blue circles)^32^ to be compared with the effect of RSV in the HGF-1 cell system: carpinontriol B (CAB),^35^ dioscin (DIOS),^36,37^ iso-alpha acids (IsoA),^38^ isosinensetin (IsoSNT),^39^l-arginine (l-Arg),^40^ and theobromine (THEO).^40^ Following a model-based normalization to account for passage and plate variations, a dose–response analysis of the individual bitter taste compounds yielded the effective 25% inhibition dose concentrations (ED25) that reduced the *Pg*-LPS-evoked IL-6 release by HGF-1 cells. Finally, a linear model of the ED25 values and the bitter taste thresholds from the literature was established and compared with results obtained from the already implemented bitter response assay based on HGT-1 cells.^27−29^

## Materials and Methods

### Chemicals

Lipopolysaccharides of *Porphyromonas gingivalis* (*Pg*-LPS) were acquired from InvivoGen. Triton X-100 solution was obtained from Carl Roth GmbH and the SuberBlock buffer from ThermoFisher. The IL-6 sandwich enzyme-linked immunosorbent assay (ELISA, ab178013) was bought from abcam. Sinensetin (SNT, 99.67% purity) and isosinenstin (IsoSNT, 99.26% purity) were purchased from MedChemExress. Quinine HCl (QHCl, 97% purity) was obtained from Combi-Blocks, and *trans*-resveratrol (RSV, 98% purity) was obtained from Breko GmbH. Epigallocatechin gallate (EGCG, 95% purity) as well theobromine (THEO, 98% purity) were purchased from Sigma Aldrich (Merck KGaA, Darmstadt, Germany). Bitter tasting compounds showing diverse and/or similar chemical properties from/to RSV were selected from the chemical space^32^ (Figure 1). The sodium salt of homoeriodictyol (3′-methoxy-4′,5,7-trihydroxyflavanone) (HED, 94% purity), carpinontriol B (CAB, 94.2% purity) and (−)-matairesinol (MAT, 95% purity) were kindly provided by Symrise AG, synthesized, and chemically characterized as described previously.^34^ The mixture of *trans*-*iso*-alpha acids was purified from a commercially available *iso*-alpha-acid extract (*iso*-extract 30%, Hopsteiner, Mainburg, Germany) following a protocol from the literature.^41^ Analysis of the isolated mixture by quantitative ^1^H NMR spectroscopy^42^ confirmed a purity of >95%, whereas targeted HPLC–MS/MS analysis verified the expected distribution across the 3 main *trans*-*iso*-alpha acids (*trans*-isocohumulone 30%, *trans*-isohumulone 40%, *trans*-isoadhumulone 30%).

All other chemicals were obtained from Sigma Aldrich (Merck KGaA, Darmstadt, Germany), unless indicated otherwise.

### Cell Culture

Human gingival fibroblasts (HGF-1; passage number 16) obtained from the American Type Culture Collection (ATCC 2014), human gastric tumor (HGT-1) attained from C. Laboisse (Laboratory of Pathological Anatomy, Nantes, Frances), and HEK 293 T-Gα16gust44 from ATCC were cultivated in Dulbecco’s modified Eagle’s medium (DMEM) high glucose (4.5 g/L d-glucose), GlutaMAX (ThermoFisher) supplemented with 10% fetal bovine serum (FBS) (Invitrogen, Vienna, Austria), 100 U mL^–1^ penicillin, and 0.1 mg mL^–1^ streptomycin at standard conditions (37 °C, 5% CO_2_, and humidified atmosphere).^9^

For cell culture studies, compounds insoluble in water were predissolved in dimethyl sulfoxide (DMSO), resulting in a final test concentration of 0.1% (v/v). Appropriate controls of cell culture medium treated cells only always contained 0.1% (v/v) DMSO. For inducing a pro-inflammatory IL-6 release, HGF-1 cells were treated with *Pg*-LPS in a final concentration of 10 μg/mL for 6 h.^7−9^

Cell viability for all treatments was proven by the MTT (3-(4,5-dimethyl thiazolyl-2)-2,5-diphenyltetrazolium bromide) assay as described previously for HGF-1 and HGT-1 cells.^9^

### Immunofluorescence Staining of GNAI3 in HGF-1 Cells

Immunofluorescence analysis of GNAI3 was performed using 70% confluent HGF-1 cells on a cover slip. The cells were washed with PBS and fixed with 3% (v/v) formaldehyde in phosphate-buffered saline (PBS) for 20 min. Next, the fixation solution was removed, and the cells were washed with PBS. Afterward, a 30 min permeabilization step of the HGF-1 cells was carried out with 1% (v/v) Triton X-100 in SuberBlock buffer and washed with PBS again. Cells were blocked with 5% (v/v) Donkey serum and 0.5% (v/v) Triton X-100 in SuberBlock buffer for 1 h. Afterward, cells were labeled with the primary antibody, GNAI3 polyclonal antibody (# PA5-27940, InvivoGen) 1:200 (v/v), in SuberBlock buffer with 5% (v/v) Donkey serum and 0.2% (v/v) Triton X-100 overnight at +4 °C. Cells were then washed and labeled with the second antibody 1:200 (v/v) goat anti-rabbit IgG H&L (Alexa Fluor 488, Thermo Fisher Scientific) preadsorbed (ab150081, abcam) for 2 h. The cells were than washed and incubated with ActinRed 555 ReadyProbes Reagent (rhodamine phalloidin) (Invitrogen), which has a high affinity to the F-actin, following the manufacturer’s protocol. Next, the cells were washed, and the nucleus was stained with NucBlue Fixed Cell ReadyProbes Reagent (DAPI) (Invitrogen) following the manufacturer′s protocol. Finally, cells were washed, embedded with anti-fade fluorescence mounting medium, Aqueous, Fluoroshield (ab104135, abcam), and analyzed by confocal laser-scanning microscopy (LSM 800 KMAT, Zeiss). As control, cells without any staining with only primary antibody and only secondary antibody labeling were analyzed.

### Selection of Test Compounds from the Bitter Chemical Space

For a first set of experiments (Figure 1, red circles), EGCG,^43^ NAR,^44^ and SNT^39^ (Table 1) were selected in order to test whether these compounds, showing a structural similarity to RSV, yet targeting a wider range of TAS2Rs as compared to RSV, demonstrate a similar or different effect on the *Pg*-LPS-induced IL-6 release in HGT-1 cells compared RSV and to QHCl^45^ (Table 1) as a structurally more divergent compound.

**Table 1:**
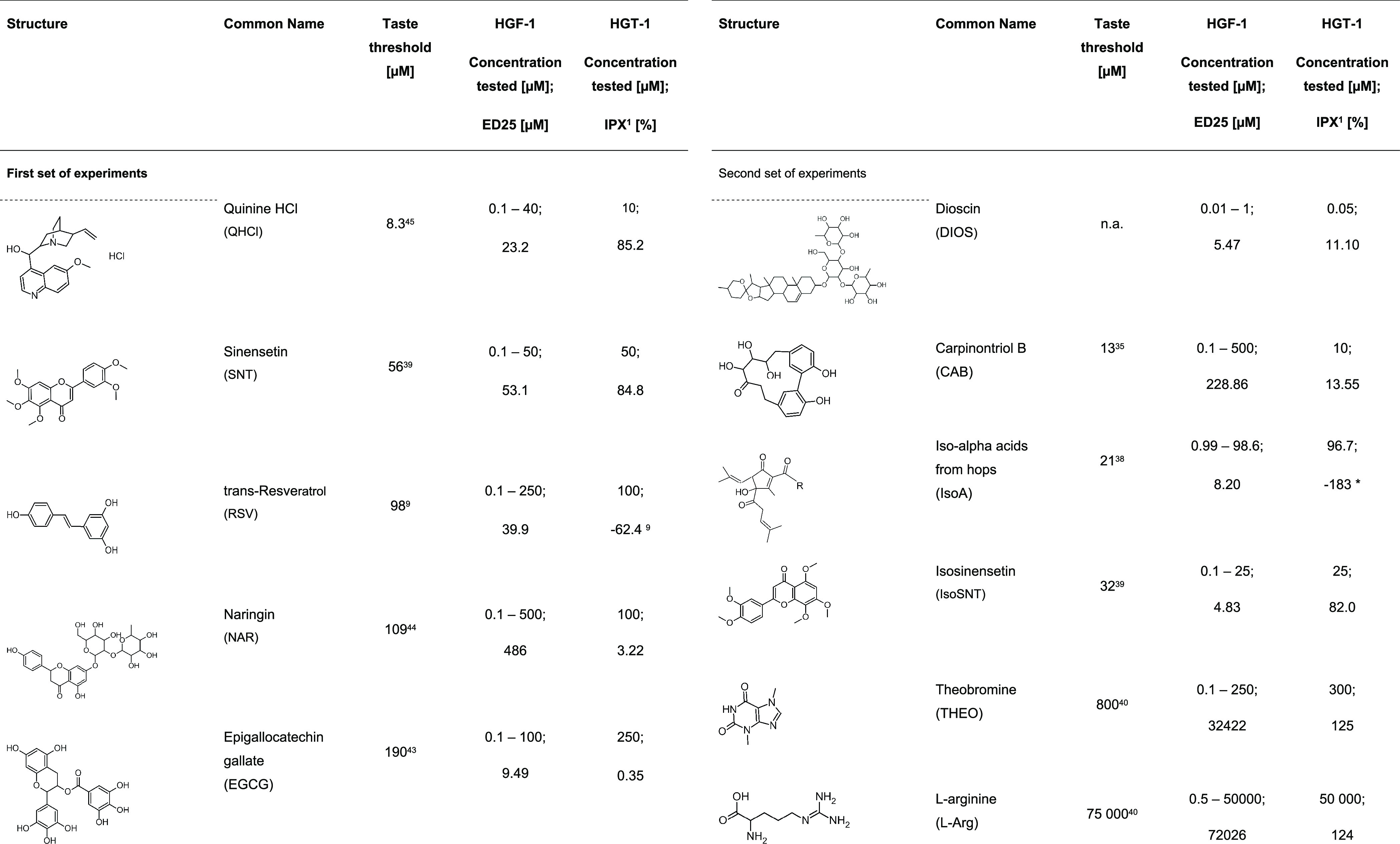
Structures, Names, Abbreviations, and Tested Concentration Range of the Selected Substances from the Bitter Chemical Space^32^^a^

a Additionally, the effective dose [μM] of bitter compound needed to reach a 25% inhibition of the 10 μg/mL *Pg*-LPS-induced IL-6 release by HGF-1 cells as well as IPX [%] values^1^ normalized to plate-dependent histamine effect of HGT-1 cells and the tested concentration of the respective substances in μM are given.

Involvement of a wider range of TAS2Rs was additionally tested by using the well-known bitter masking compounds HED^29,34^ and MAT,^29^ targeting TAS2R20/31/43/50 ^9, 27^ and TAS2R4 and 43, respectively. In a second set of experiments, we investigated a diverse subset of compounds spanning over a larger area of the chemical space^32^ that target a wider range of TAS2Rs: CAB,^35^ DIOS,^36,37^ Iso-alphaA,^38^ IsoSNT,^39^l-Arg ^40^and THEO^40^ (Figure 1, blue circles, Table 1).

At least six concentrations per compound with two technical and three to seven biological replicates were tested in the cell assays, covering literature taste threshold concentrations from sensory studies (Table 1).

### Quantitation of IL-6 Release by HGF-1 Cells by Means of Enzyme-Linked Immunosorbent Assay (ELISA)

HGF-1 cells were seeded in 96 well plates at a density of 5000 cells per well 72 h before the experiment. Prior to addition of 10 μg/mL *Pg*-LPS, the cells were pretreated for 1 h with the selected bitter substance from the chemical space (Figure 1, red circles), EGCG (100 μM), NAR (500 μM), QHCl (40 μM), and SNT (50 μM) with or without HED or MAT in a concentration ratio of 10:1.^18,20^ After exposure, the cells’ supernatants were harvested for the ELISA analysis after an incubation time of 6 h. In a second set of experiments, CAB, DIOS, EGCG, IsoA, IsoSNT, l-Arg, NAR, QHCl, RSV, SNT, and THEO (Figure 1, blue circles) in concentration ranges that covered their individual bitter taste threshold concentration (Table 1) were tested as described above. The supernatant was then applied to a sandwich enzyme-linked immunosorbent assay (ELISA, ab178013) to quantitate the IL-6 release of HGF-1 cells (abcam, minimal detectable dose (MDD): 1.6 pg/mL, intra-assay reproducibility CV 2.1%, inter-assay reproducibility CV 2.4%), following the manufacturer’s protocol. The optical density (OD) at 450 nm was measured by a Tecan Infinite M200 PRO plate reader (Tecan, Switzerland).

### Molecular Docking

Molecular docking simulations were run to deduce the potential binding pose of SNT, EGCG, QHCl, and NAR, into the 3D structure of TAS2R50, as modeled previously.^9^ The ligands were prepared for the docking with the generation of stereoisomers and protonation states at pH 7 ± 0.5 with LigPrep, as implemented in the Schrödinger Small-Molecule Drug Discovery Suite Schrödinger Release 2021-4 (Schrödinger, LLC, New York, NY, 2021). Molecular descriptors AlogP, hydrogen bond acceptors (HBAs), and hydrogen bond donors (HBDs) were calculated with Schrödinger Release 2021-4 (Schrödinger). Because of the different molecular size of the investigated compounds and the uncertainness of the modeling of the extracellular loop 2 and 3 (ECL2 and 3), we cut the model from residue Asp150^ECL2^ to Arg169^5.32^ and from Trp250^6.60^ to Pro259^7.33^, respectively. Glide Standard Precision was used for docking simulations. The coordinates of Trp88^3.32^ were used as centroid during the Receptor Grid Generation (Schrödinger Release 2021-4: Glide, Schrödinger). Three rotatable groups (Ser248^6.58^, Tyr85^3.29^ and Tyr176^5.39^) were allowed to move. Docking poses underwent molecular mechanics generalized Born surface area (MM-GBSA) calculations (Schrödinger Release 2021-4: Prime, Schrödinger) (sampling method, minimize).

### Analysis of ED25 Values and Establishment of a Linear Model

Normalization of uncorrected ELISA absorbance data was performed by a model-based approach using the plate and passage specific effect of the addition of a fixed concentration of RSV 100 μM to *Pg*-LPS treated cells. For each combination of passage and plate, individual linear models describing the absorbance signal reduction between *Pg*-LPS treated HGF-1 cells and similar cells treated in addition with RSV (100 μM, *n*_technical replicates_ = 2, and *n*_biological replicates_ = 3–6) were trained. Correction factors for each plate and passage were obtained from the model parameters in contrast to the global model incorporating the complete data and subsequently applied to the raw data.

Dose–response analysis of individual bitter agonists were carried out using the normalized data and extension package drc (version 3.0–1) for the statistical programming environment R and the built-in four-parameter log-logistic model functions. Dose–response models were estimated for the tested concentration ranges based on the obtained function parameters, visualized using the ggplot2 R package (version 3.3.6) and followed by revealing 25% effective inhibitory concentrations to correlate with the compounds’ bitter taste threshold concentrations.

### Quantitation of the Intracellular Proton Index (IPX) in HGT-1 Cells

HGT-1 cells were seeded in a density of 100,000 cells per well in 96 well plates 24 h prior to the measurement. Following cell viability analyses carried out in transparent 96 well plates, proton secretion assays for determining the intracellular proton index (IPX) were performed in black 96-well plates.

Using HGT-1 cells, the intracellular pH (pH_i_) was determined by means of the pH sensitive fluorescence dye SNARF-1AM for the investigation of the proton secretion, as described before.^27,46^ Briefly, HGT-1 cells were cultivated and seeded 24 h before the experiment. Cells were washed with 100 μL Krebs-Ringer-HEPES buffer (KRHB; 10 mM 4-(2-hydroxyethyl)-1-piperazineethane-sulfonic acid (HEPES), 11.7 mM d-glucose, 4.7 mM KCl, 130 mM NaCl, 1.3 mM CaCl_2_, 1.2 mM MgSO_4_, and 1.2 mM KH_2_PO_4_, adjusted to a pH of 7.4 with 5 M KOH) per well, and stained with 3 μM 1,5-carboxy-seminaphtorhodafluor acetoxymethyl ester (SNARF-1 AM) for 35 min at standard conditions. After the staining, the HGT-1 cells were washed twice with 100 μL of KRHB per well, and 100 μL of the investigated compound and the positive control histamine (1 mM) were added to the cells and incubated for at least 10 min. For substances, which are interfering with the fluorescence signal because of their own color, an additional washing step was performed after the incubation with the substance. Fluorescence was measured at 580 and 640 nm emission after excitation at 488 nm using a Flexstation 3 (Molecular Devices, Sunnyvale, CA, USA). The IPX was determined by using a pH-calibration curve (pH 7.0–8.0), calculating the intracellular H^+^ concentration, followed by the calculation of a ratio between the treated and untreated cells. Data calculation was performed with a log2 of the ratio of treated to untreated cells, revealing the intracellular proton index (IPX), with a high secretory activity indicated by a low IPX-value. For the correlation analysis, the data were normalized to the respective histamine response.

### Data Analysis

Pathway analysis for elucidation of a possible connection between TAS2R50 activation and IL6 release was performed using the pathway topology analysis tool integrated into the reactome database.^47^ To analyze the connectivity, all proteins included in the interleukin-6 family signaling pathway (ID: R-HSA-6783589.6; DOI 10.3180/r-hsa-6783589.1) for species *Homo sapiens* as well as the bitter taste receptor TAS2R50 were imported into the Gene Set Analysis Function of the Reactome FIViz plugin in Cytoscape (Reactome FI Network Version 2021). Analysis was conducted using the linker genes option to integrate genes required for connection of the individual pathways. Visualization of the resulting gene set and connectivity network was performed by means of Cytoscape (version 3.9.1, cytoscape.org).

Statistical analysis was performed using the statistical programming environment R (version 4.2.0). Model assumptions were evaluated using the Shapiro–Wilk test of normality and Levene’s test for homogeneity of variance across groups implemented in the R package car. Nonparametric comparison of the location parameters of multiple groups was performed by the Kruskal–Wallis rank sum test, while the procedure described by Siegel and Castellan (1988)^48^ was applied for subsequent multiple comparison tests using the kruskalmc function implemented in the pgirmess package.

## Results

### Pathway Analysis of IL-6 and TAS2R50 and the Proof via Immunofluorescence Staining

The pathway analysis revealed GNAI3 as an upstream signaling protein that links TAS2R50 and the IL-6 pathway via phosphatidylinositol 4,5-bisphosphate 3-kinase catalytic subunit alpha isoform (PIK3CA), nuclear factor NF-kappa-B (NfKB1) and AP-1 subunit, and the heterodimer Fos-Jun (Figure S1A). We, therefore, verified the protein expression of GNAI3 in the HGF-1 cells studied by immunofluorescence staining (Figure S1B). The fluorescence signal was GNAI3 specific and confirmed our pathway analysis, whereas cells treated with the secondary antibody solely did not show a specific signal (Figure S1B).

### Effect of the TAS2R Agonists SNT, EGCG, QHCl, and NAR on *Pg*-LPS Induced IL-6 Release in HGF-1 Cells

In order to ensure that none of the test compounds had a detrimental effect on the viability of HGF-1 cells, an MTT-test was conducted prior to the IL-6 release experiments. The results revealed no effect on the cellular viability by any of the test compounds in any of the concentrations tested (Table S2).

First, the effect of the bitter tasting non-TAS2R50 agonists QHCl^19^ and EGCG^18,49^ as well as the bitter tasting NAR and SNT, for which no TAS2R activation has been tested so far, on the IL-6 release evoked by *Pg*-LPS in HGF-1 cells was tested at concentrations of 40, 50, 100, and 100 μM, respectively. Individual test concentrations were selected from preceding dose–response experiments, in which those concentrations showed the maximum effect size (Figure S2).

**Figure 2:**
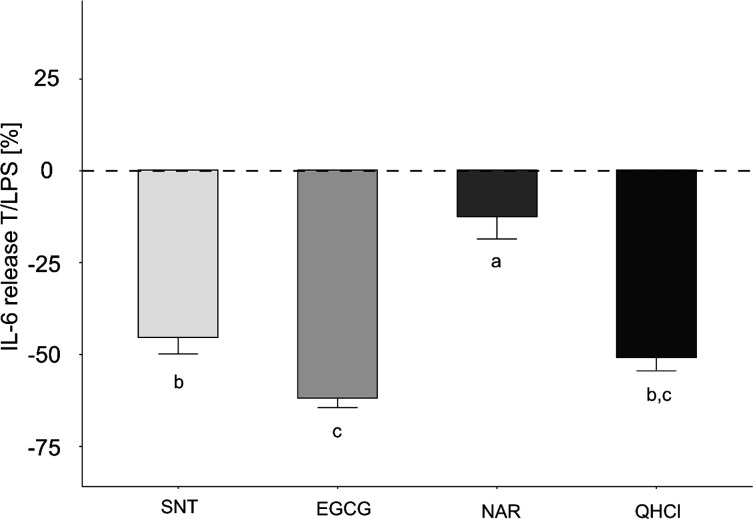
IL-6 release of HGF-1 cells treated with SNT 50 μM, EGCG 100 μM, NAR 100 μM, and QHCl 40 μM in a 1 h preincubation, followed by a 6 h co-incubation to *Pg*-LPS. Treatment of HGF-1 cells with 10 μg/mL *Pg*-LPS for 6 h solely revealed a mean IL-6 release of 42.62 ± 3.35 pg/mL (*n* ≥ 4; t.r. = 2). Data displayed are calculated as treatment over control in percent, with the effect of *Pg*-LPS set to zero (T/LPS, %; LPS = 0%). Data are shown as mean ± SEM *n* = 3–4; t.r. = 2. Statistics: Kruskal–Wallis; *p* ≤ 0.05, post hoc test: multiple comparison tests using the kruskalmc function.

The resulting mean inhibitory effect sizes were −12.6 ± 5.92% for NAR, −45.5 ± 4.28% for SNT, −51.0 ± 3.42% for QHCl, and −62.1 ± 2.37% for EGCG (all *p* ≤ 0.05, Figure 2A). At these concentrations, the effect of QHCl did only differ from that demonstrated for NAR, whereas no statistical difference was found when the results for QHCl were compared with those for SNT and EGCG.

### Putative Binding Mode of SNT, NAR, EGCG, & QHCl to TAS2R50

To investigate the potential interaction of SNT, NAR, EGCG, and QHCl to the TAS2R50 binding site, molecular docking simulations were performed. Because of the uncertainness of ECL2 modeling and the different molecular sizes of the compounds (Table S1), the ECL2 region was removed from the model.^50−53^ The ECL2 connects transmembrane helices 4 and 5 and is, except for a conserved Asn-linked glycosylation site,^54^ the longest and most diverse loop among TAS2Rs.^55^ It was demonstrated that docking performance could be insensitive to or even improved by excluding ECL2 from the calculations.^50−52^ Indeed, the model without the ECL2 and ECL3 was proven to be able to reproduce the docking pose of RSV (Figure S3), as previously published.^9^

Among the analyzed compounds, SNT, with a docking score of −7.54 kcal/mol, is the compound predicted to best interact with TAS2R50 and is the one that most closely reproduces the RSV binding mode. On the contrary, EGCG and QHCl have low docking scores, thus confirming a lack of robust interaction of these compounds to TAS2R50 (Figure S3).

### Effect of SNT, EGCG, QHCl, NAR, and TAS2R Antagonists MAT and HED on the *Pg*-LPS Induced IL-6 Release in HGF-1 Cells

Prior to all experiments, effects on the viability of HGF-1 cells were excluded by MTT-tests performed (Table S2). In subsequent experiments, the antagonistic effect of MAT on TAS2R4 and TAS2R43 was demonstrated in HEK-293 T Gα16gust44 cells by means of a well-established Ca^2+^-mobilization assay^19,22,56^ (Figure S4). In these experiments, a concentration of 10 μM MAT reduced the effect evoked by aristolochic acid, targeting TAS2R43,^19^ and by colchicine, targeting TAS2R4,^19^ from 100% to 78.9 ± 13.3% (*p* ≤ 0.01) and to 48.9 ± 32.8% (*p* ≤ 0.05), respectively.

**Figure 3:**
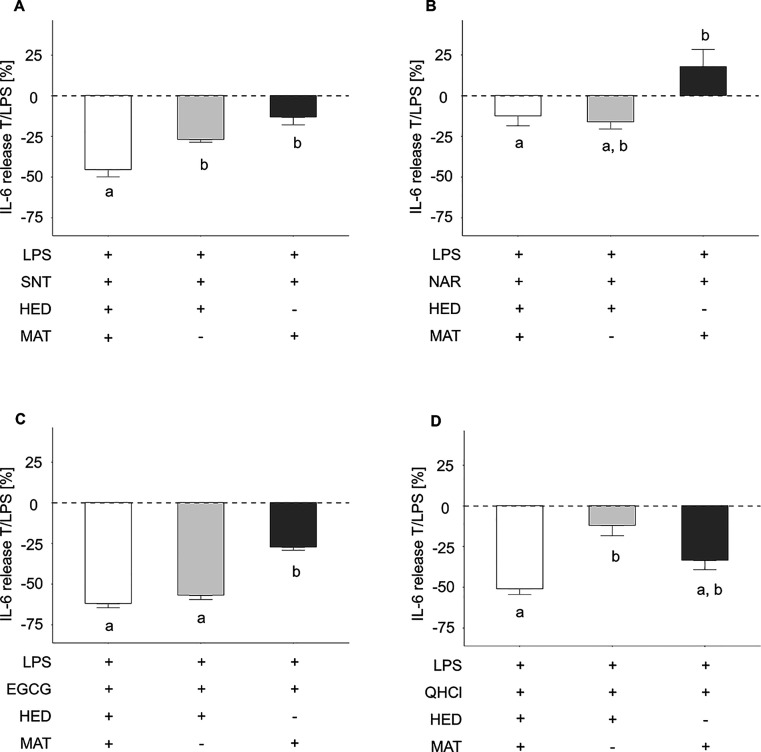
IL-6 release of HGF-1 cells treated with (A) SNT 50 μM, (B) NAR 500 μM, (C) EGCG 100 μM, and (D) QHCl 40 μM in a 1 h preincubation and a 6 h co-incubation with *Pg*-LPS, followed with or without HED or MAT treatment (10:1). Treatment of HGF-1 cells with 10 μg/mL *Pg*-LPS for 6 h solely revealed a mean IL-6 release of 42.62 ± 3.35 pg/mL (*n* ≥ 4; t.r. = 2). Data displayed are calculated as treatment over control in percent, the effect of *Pg*-LPS set to zero (T/LPS, %; LPS = 0%). Data are shown as mean ± SEM *n* = 3–4; t.r. = 2. Statistics: Kruskal–Wallis; *p* ≤ 0.05, post hoc test: multiple comparison tests using the kruskalmc function.

Involvement of multiple TAS2Rs in the repressive effect of bitter tasting food constituents on the *Pg*-LPS evoked IL-6 release in HGF-1 cells was also tested in co-incubation experiments with MAT and HED (Figure 3). Whereas MAT reduced the *Pg*-LPS evoked IL-6 release of SNT, NAR, and EGCG by mean percentage effect sizes of −27.1 ± 1.60, −17.8 ± 10.6, and −27.4 ± 1.77% (all *p* ≤ 0.05, Figure 3A–C), respectively, HED demonstrated a reducing effect when co-applied together with SNT (−13.2 ± 4.67%, *p* ≤ 0.05, Figure 3A) and QHCl (12.1 ± 6.28% *p* ≤ 0.05, Figure 3D).

### Association between the Repression of *Pg*-LPS Induced IL-6 Release in HGF-1 Cells by Compounds from the Chemical Bitter Space and Their Bitter Threshold Concentration

**Figure 4:**
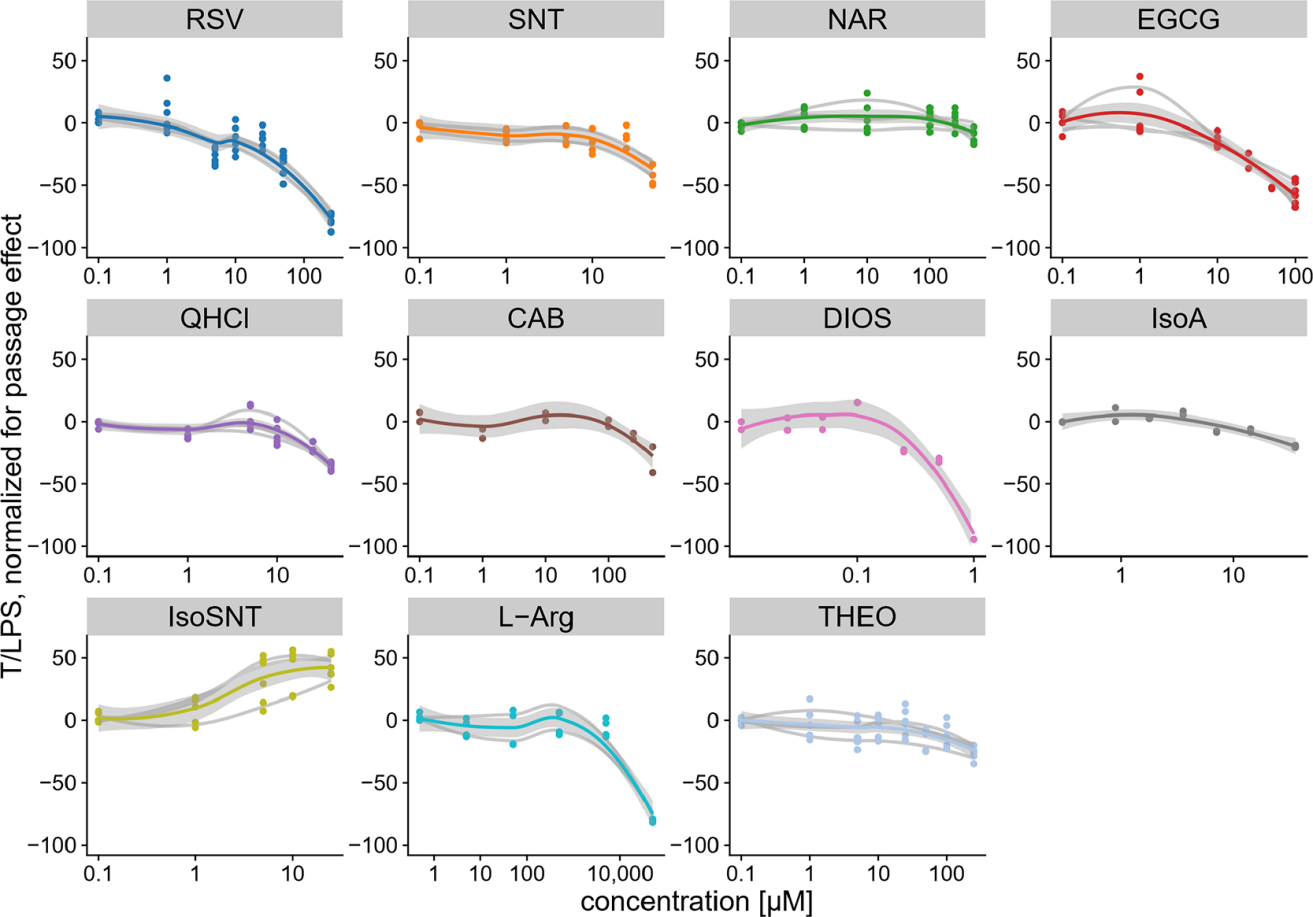
Dose response curves recorded for CAB, DIOS, EGCG, IsoA, IsoSNT, l-Arg, NAR, QHCl, RSV, SNT, and THEO at concentration ranges from 0.1 μM to 50 mM, covering the taste threshold values, after 1 h preincubation followed by a 6 h co-incubation with *Pg*-LPS. Treatment of HGF-1 cells with 10 μg/mL *Pg*-LPS for 6 h solely revealed a mean IL-6 release of 35.49 ± 1.53 pg/mL (*n* ≥ 4; t.r. = 2). Data displayed are normalized for passage and plate effect of the cells’ response to a fixed RSV 100 μM concentration (*n* = 3–6; t.r. = 2).

**Figure 5:**
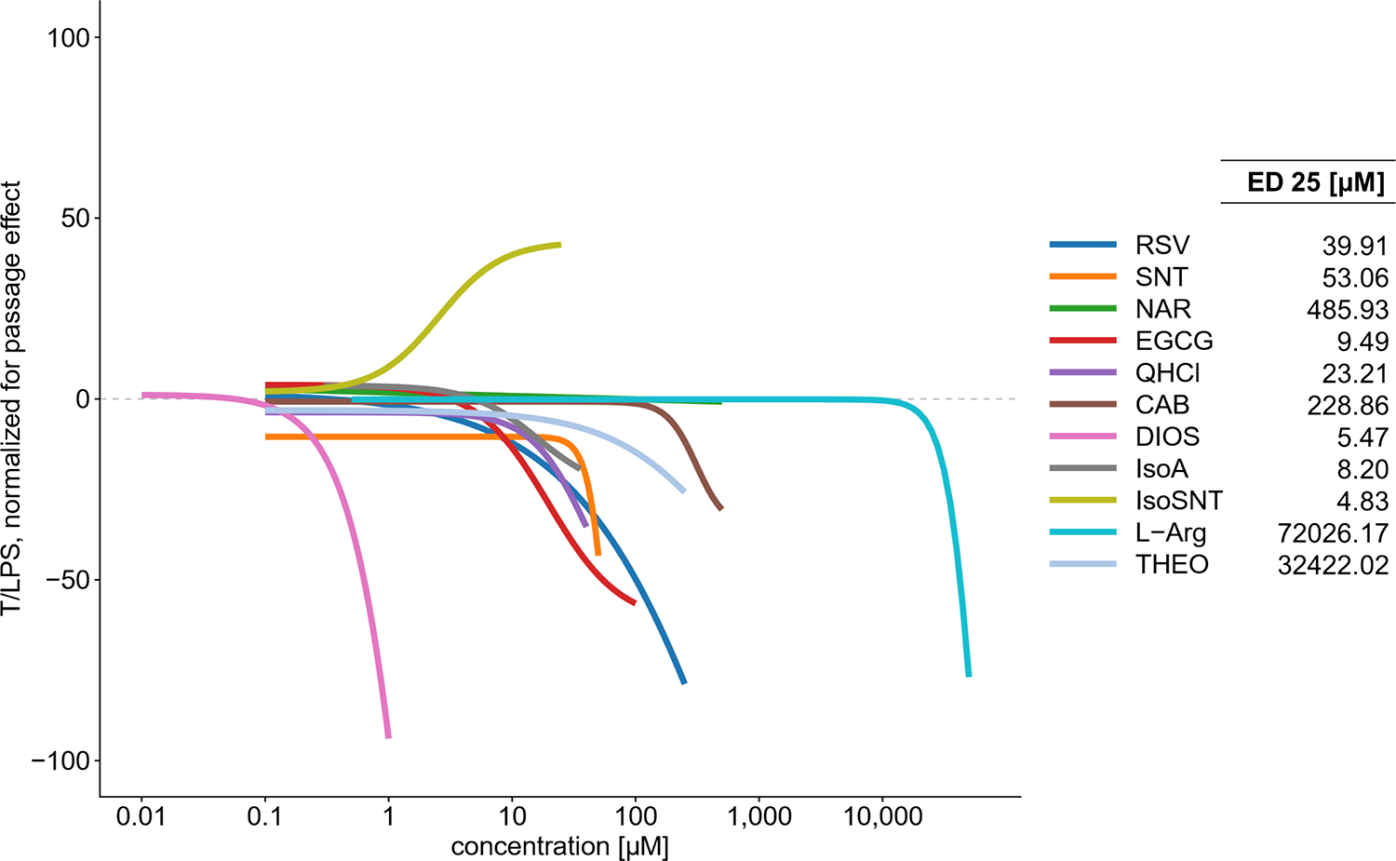
IL-6 release dose response of HGF-1 cells treated with CAB, DIOS, EGCG, IsoA, IsoSNT, l-Arg, NAR, QHCl, RSV, SNT, and THEO in a 1 h preincubation, followed by a 6 h co-incubation with *Pg*-LPS. Treatment of HGF-1 cells with 10 μg/mL *Pg*-LPS for 6 h solely revealed a mean IL-6 release of 35.49 ± 1.53 pg/mL (*n* ≥ 4; t.r. = 2). Data displayed are normalized for passage effect and calculated as treatment over *Pg*-LPS (T/LPS, LPS = 0) (*n* = 3–6; t.r. = 2).

To test whether a repressive effect on the *Pg*-LPS induced IL-6 release by HGF-1 cells is associated with a compounds’ bitter taste threshold concentration published in the literature, effects of the following test compounds from the chemical bitter space were investigated in dose response experiments: CAB, DIOS, EGCG, IsoA, IsoSNT, l-Arg, NAR, QHCl, RSV, SNT, and THEO (Figure 1). Test concentrations applied covered the individual compounds’ bitter taste threshold concentration published, spanning over a total concentration range of 0.1 μM–50 mM (Figure 4, Table 1). After normalization for passage and 96-well plate effects, all test compounds except IsoSNT showed a dose-dependent repressing effect on the *Pg*-LPS induced IL-6 release (Figure 4). Next, individual data was used to calculate an effective inhibitory dose of 25% (ED_25_) for each compound (Figure 5). The ED_25_ values for CAB (228.86 μM), DIOS (5.47 μM), EGCG (9.49 μM), IsoA (8.20 μM), IsoSNT (4.83 μM), l-Arg (72026.17 μM), NAR (485.93 μM), QHCl (23.21 μM), RSV (39.91 μM), SNT (53.06 μM), and THEO (32422.02 μM) were correlated with the respective bitter taste threshold concentration of 13 μM for CAB,^35^ 190 μM for EGCG,^43^ 21 μM for Iso-A,^38^ 32 μM for IsoSNT^39^, 75,000 μM for l-Arg,^40^ 109 μM for NAR,^44^ 8.3 μM for QHCl,^45^ 98 μM for RSV,^9^ 56 μM for SNT,^39^ and 800 μM for THEO^40^ published in the literature (Table 1). This linear correlation analysis according to Pearson revealed a correlation coefficient of *R*^2^ = 0.599 (*p* = 0.014) was achieved. Notably, DIOS was excluded from this analysis because no taste threshold data were available in the literature.

### Comparison of the Bitter Response in the HGF-1 Cell Model with the Bitter-Taste Associated Changes of the Intracellular Proton Index in HGT-1 Cells

**Figure 6:**
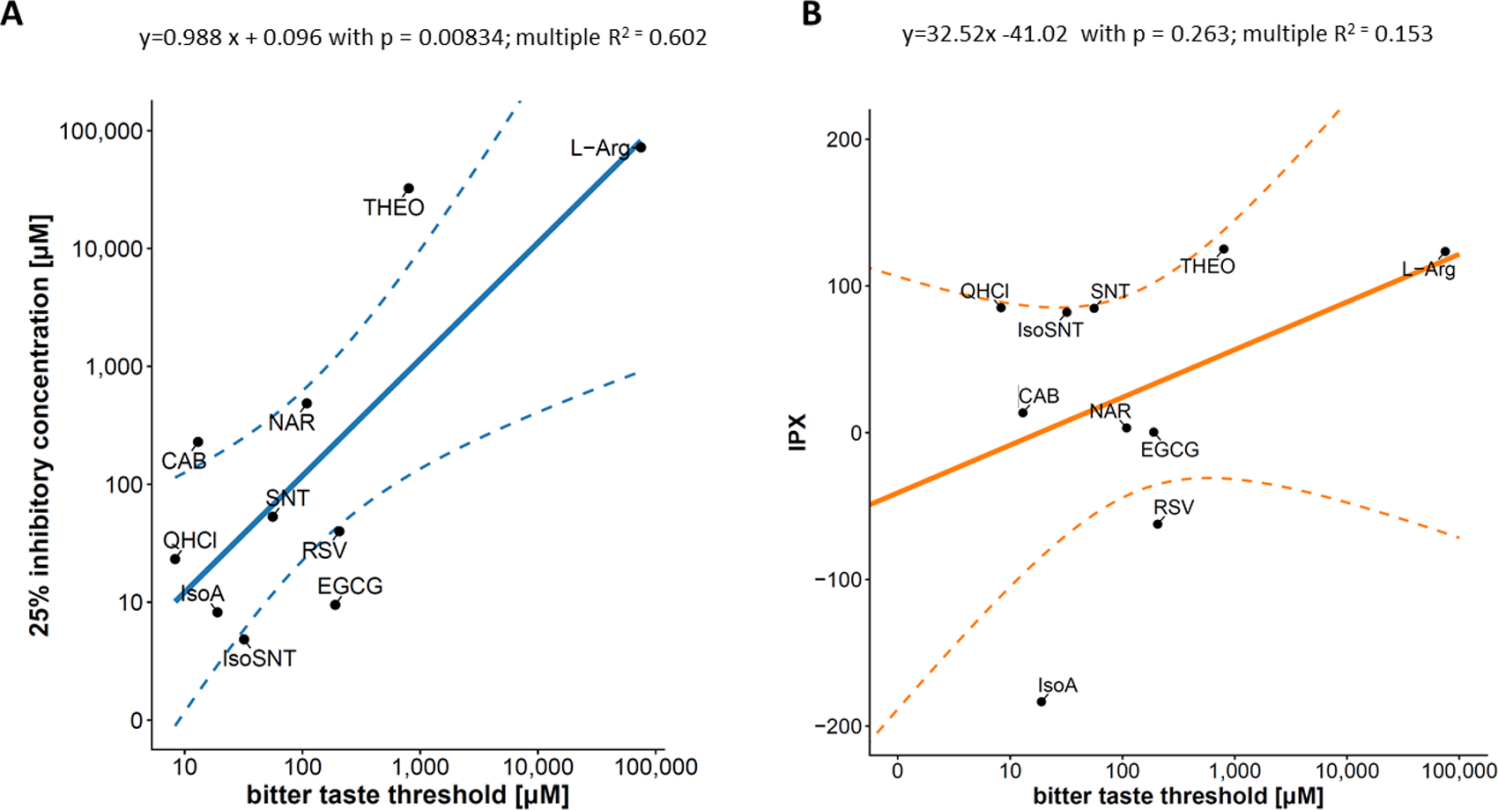
(A) Pearson correlation of the 25% inhibitory concentration [μM] of the selected bitter tasting compounds in HGF-1 with their respective bitter taste thresholds [μM] log transformed: *R*^2^ = 0.602, *p* = 0.008. (B) Pearson correlation of IPX-values in HGT-1 cells normalized to the respective histamine control and the tested concentrations of the bitter tasting compound [μM] log transformed: *R*^2^ = 0.153, *p* = 0.263.

In order to compare the linear bitter response model established for the HGF-1 cells with our previously published HGT-1 cell model,^27,29^ all test compounds of the here-presented study were also tested for their impact on the intracellular proton index (IPX) in HGT-1 cells as an indicator of bitter taste quality.^27,29^ Test concentrations covered the taste threshold concentrations (Table 1). After normalization of the mean IPX effect to the stimulating effect of histamine as internal quality control, mean effect sizes for CAB (13.55 ± 53.23%), DIOS (11.10 ± 35.35%), EGCG (0.35 ± 69.94%), IsoA (−183.42 ± 30.59%), IsoSNT (82.04 ± 37.84%), l-Arg (123.49 ± 156.85%), NAR (3.22 ± 44.11%), QHCl (85.17 ± 164.02%), RSV (−62.39 ± 100.96%), SNT (84.76 ± 50.58%), and THEO (125.15 ± 112.11%) were revealed. In analogy to the linear model established for the HGF-1 cells (Figure 6A), the association between the bitter threshold concentration (Table 1) and the mean IPX was investigated and revealed no significant linear relationship (*R*^2^ = 0.153, *p* = 0.263).

## Discussion

Mammalian TAS2Rs are expressed in several extra-gustatory tissues, e.g., the upper airways,^57,58^ lungs,^59^ gastrointestinal tract,^27,60^ brain,^61^ bladder,^62^ and testis,^63^ as well as immune competent cells, such as blood monocytes and leukocytes^64^ and gingival fibroblasts.^9,10^ For gingival fibroblasts, Zhou et al.^10^ recently published the protein expression of guanine nucleotide-binding protein G subunit alpha 3 (GNAT3), an upstream GPCR signaling protein, and provided results that suggest an involvement of TAS2Rs in cellular downstream inflammatory responses.^10^ In the here-presented work, the HGF-1 cells studied were stained positive for GNAI3, thereby confirming the presence of another signaling subunit in addition to GNAT3 reported by Zhou et al.^10^ In recent works, it has been shown that in stem cells of the apical papilla (SCAPs), GNAI3 plays an important role in mineralization and tissue regeneration via the c-Jun N-terminal kinase (JNK) and the extracellular-signal regulated kinase (ERK) signaling.^30^ For HGF cells, presenting the predominant cell type in gingival connective tissue, GNAI3 is known to promote wound healing by promoting the generation of an extracellular matrix. Moreover, in mice, it was shown that GNAI1 and GNAI3 suppress GNAI2-mediated colitis-associated tumorigenesis through negatively regulating Janus kinase (JAK) and NF-κB promoted IL-6 signaling.^65^ According to existing pathway data from reactome, we deem the pathway shown in Figure S1 to be relevant for the TAS2R-induced intracellular signaling resulting in IL-6 gene transcription via NFκ-B. Moreover, the work by Zhou et al.^10^ showed that heterotrimeric G-protein subunits PLCβ2 and GNAT3 are relevant in HGF-1 cells for their response to LPS. In this study, we additionally demonstrated that GNAI3, also belonging to the G-protein family, is functionally expressed in HGF-1 cells. Therefore, we consider the TAS2R-induced G-protein signaling as relevant for the cellular IL-6 response.

In the context of mammalian innate immune responses to environmental stimuli, such as metabolites derived from bacteria (e.g., LPS or quorum-sensing molecules), extra-gustatory TAS2Rs have been hypothesized to function as immune sentinels.^58,66,67^ Also, more specifically, TAS2R activation in human whole blood and lung macrophages^59,68^ as well as gingival fibroblasts^9,10^ has been shown to antagonize LPS-induced pro-inflammatory cytokine production, suggesting a potential role of TAS2Rs in the control of inflammation.

Supported by recently published TAS2R pathway analyses^10^ and our own results presented here, demonstrating TAS2R downstream signaling proteins being linked to the release of immune-modulatory, inflammatory cytokines in HGF-1 cells, we hypothesized the HGF-1 cell line expressing almost all *TAS2R*s^10^ as a novel screening tool for the identification of bitter tasting and bitter taste modulating compounds.

Since our previous study in HGF-1 cells revealed TAS2R50 to mediate the RSV-evoked reducing effect on the IL-6 release induced by *Pg*-LPS,^9^ we also aimed to elucidate in the study presented here, whether this IL-6 response depends on TAS2R50 specifically or whether activation of a wide range of TAS2Rs is associated with this cellular response. To answer this question, four bitter tasting compounds (EGCG, NAR, QHCl, and SNT)^58,59,61^ that are structurally similar or divergent from RSV were selected from the chemical bitter space and subjected to a bitter-sensing HGF-1 cell assay, which was paired with a chemoinformatic modeling approach.

Results from the HGF-1 cell assay revealed no clear indication for a specific involvement of TAS2R50, since all tested compounds induced a cellular response, as previously reported for RSV targeting TAS2R50.^9^ The specific involvement of TAS2R50 could also be excluded by the results from docking studies, since we did not find a consensus in the binding modes of the four compounds to TAS2R50. In some cases, as for QHCL and EGCG, the docking scores were very low, whereas the response in the HGF-1 cell assay was strong, indicating no specific role of TAS2R50 in the repression of a *Pg*-LPS-induced IL-6 release by HGF-1 cells. Application of the bitter masking compounds HED,^29,34^ targeting TAS2R20/31/43/50,^27^ and MAT, demonstrated to target TAS2R4/43, indicate that likely more TAS2Rs than only TAS2R50 are involved in the cellular response studied. Depending on the agonist applied, co-treatment of the HGF-1 cells with one of the antagonists HED or MAT resulted in different cellular responses induced by the individual agonists EGCG, QHCl, NAR, and SNT, with each of them targeting multiple TAS2Rs (EGCG: TAS2R4/5/14/30/39/43;^18,69,70^ QHCl: TAS2R4/7/10/14/31/39/40/43/46;^19^ NAR: TAS2R not known yet; SNT: TAS2R not known yet). We hypothesize these results to demonstrate a complex interplay of several TAS2Rs, resulting in a net modulation of the cells’ IL-6 release in response to *Pg*-LPS. This hypothesis is supported by our previously established screening model founded on the native cell line HGT-1, in which activation of TAS2Rs results in the secretion of protons.^27^ In this cell model, application of bitter tasting and bitter taste masking compounds results in a modulation of proton secretion, calculated as the intracellular proton index (IPX), which correlates well with the sensory bitter taste intensity.^29^ However, the HGT-1 cell assay presents some limitations since results were rarely not in line with those obtained from sensory trials, e.g., when phloretin^29^ or other food compounds that quench the fluorescence of membrane-bound probes^71^ were tested. Also, testing of pH-sensitive compounds^72^ and the requirement of high-throughput testing are restricting factors for this cell model.^73^ None of these limitations applies to the HGF-1 cell assay presented here: the ELISA technique is less sensitive to quenching of the test compounds, does not rely on intracellular changes of the pH and is based on a robust technique that can easily be transformed into a high-throughput format.

In order to verify an association between a compound’s ability to modulate the *Pg*-LPS-induced IL-6 release by HGF-1 cells and its bitter taste threshold concentration, a linear model was established. This model is founded on the literature of bitter taste threshold data and dose response curves of 11 bitter tasting, chemically diverse food constituents spanning over the chemical bitter space,^32^ with each compound tested in at least six concentrations in three to four independent experiments with two technical replicates. For all of the bitter tasting compounds tested, a repressing effect on the *Pg*-LPS induced IL-6 release was demonstrated, except of Iso-SNT. One may speculate that this opposite effect could rely on the interaction with other TAS2Rs or the establishment of a different type of interaction with common target TAS2Rs (e.g., agonistic-antagonistic, agonistic-inverse agonistic, etc.).

Nevertheless, after normalization of the data for the plate and passage specific effects, a linear model using the ED_25_ was established. This model confirms a significant linear relationship with a Pearson correlation coefficient of 0.602 (*p* = 0.008). The same approach was applied to the HGT-1 proton secretion assay (IPX normalized to the histamine effect), revealing no significant relationship (*p* = 0.263) between the effect of the bitter tasting compounds on the IPX and the compounds’ bitter taste threshold. Comparing both cell assays, the HGF-1 model was demonstrated to show a better association with the bitter taste threshold than the HGT-1 model, whereas results of the HGT-1 cell model correlated well with a compounds’ bitter taste intensities.^29,46^ However, for both cell models, an involvement of TAS2R-independent pathways cannot be excluded.

Generally speaking, the overall concept of using native cell lines as screening models is well-established, since the implementation of taste receptors in cell-based screening assays provides an alternative option for the identification of novel taste modifiers in addition to sensory approaches.^26^ Alternative cell-based assays are founded on heterologous, reporter cell systems and used for the screening and validation of novel receptor ligands. One of the most commonly used cell line for this purpose is the human embryonic kidney cell line HEK-293 or variants thereof, which does not express bitter taste receptors natively and allows the recombinant expression of TAS2Rs^74^ and promiscuous G-proteins.^75^ In this cell model, agonist-induced TAS2R activation leads to an intracellular calcium mobilization, which is detected by calcium-sensitive fluorescent probes. This approach provides stable and accurate readouts in high-throughput measurements.^74^ Whereas in many cases these assays correlate well with human sensory data (e.g.,^20−25^), for some compounds, deviations between in vivo and in vitro data were found (e.g.^38,76^). The apparent differences could be due to, e.g., multiple or perireceptor events, the use/existence of different receptor variants in the in vitro assay compared to the genetic background of sensory panelists, solubility issues of some compounds, or yet unknown technical issues with those TAS2Rs that have remained orphan to date. Nevertheless, TAS2R transfected HEK-293 cells are well established to study receptor binding kinetics and to identify allosteric modulators of individual TAS2Rs.^19^

Native cell-based assays are based on immortal human cell lines from nongustatory tissue, which endogenously express TAS2R receptor genes and native taste transduction signaling pathways, thereby providing the mechanistic basis for a correlation between the sensory perception and the cellular response to ligand-receptor activation. Correlations with sensory data are often strong, since bitter compounds or bitter modulators may target several TAS2Rs,^77^ with the overall interaction of all targeted receptors generating the physiological response of bitter reception. In previous studies of our group, we established a cellular screening approach for high-intensity bitter compounds that is based on the TAS2R-mediated proton secretion by human parietal cells of the HGT-1 cell line.^29^ However, for native cell-based models targeting the identification of bitter tasting or bitter taste modulating compounds, screening models validated for bitter taste threshold data published for a wide range of compounds from the chemical bitter space are lacking.

The term “taste threshold” was introduced in the 19th century by psychophysicists and used to denote a stimulus concentration, above which the stimulus could be detected and below which it could not. According to the ISO 13301:2018(E),^78^ taste threshold evaluation studies are mainly carried out for two reasons: as measures of the sensitivity of assessors or groups of assessors to specific stimuli and as measures of the ability of substances to evoke sensory responses in assessors. In the first case, the value of the threshold is taken as a description of an assessor’s performance and in the latter, as a measure of a property of the substance. For the here-presented work, published bitter taste threshold data of selected bitter tasting compounds, reported by a wide variety of panelists and captured in the chemical bitter space,^17,32^ were associated with the compounds’ ability to reduce the *Pg*-LPS-reduced IL-6 release by HGF-1 cells. This cell model presents an alternative native cell-based assay suitable for the identification of bitter tasting food constituents, showing a linear association between a compound’s bitter taste threshold and its 25% inhibitory concentration on the IL-6 response induced by *Pg*-LPS. However, taste threshold data should always be determined by sensory studies, although cell-based assays present valuable screening approaches to identify bitter tasting and bitter taste modulating compounds. In addition, our results pinpoint a possible predictive model for the identification of bitter tasting compounds with a potential to act as anti-inflammatory substances due to their bitter taste.

Although the mechanisms and biological significance of TAS2Rs-induced anti-inflammatory effects have not been well studied in a clinical context yet, there is growing evidence for their essential role in controlling inflammatory processes, as indicated by a recently published meta-analysis of the anti-inflammatory potential of bitter tasting phytochemicals.^79^

## Supporting Information Available

Calculated molecular descriptors of EGCG, IsoSNT, NAR, QHCl, RSV, and SNT (Table S1), HGT-1 and HGF-1 cell viability after treatment of bitter tasting test compounds (Table S2), network of TAS2R50 and IL-6 pathways by reactom as well as results from immunofluorescence analysis of GNAI3 in HGF-1 cells (Figure S1), results from dose–response experiments of SNT – sinensetin (0.1–50 μM), NAR – naringin (0.1–500 μM, EGCG – epigallocatechin gallate (0.1–100 μM), and QHCl - quinine HCl (0.1–40 μM) (Figure S2), 3D representation of the docking poses of RSV, NAR, SNT, EGCG, and QHCl into the TAS2R50 binding pocket (Figure S3), and results on the antagonistic effect of 10 μM MAT in TAS2R4 and TAS2R43 transfected HEK – 293 T-Gα16gust44 cells by co-administration with TAS2R43 agonist aristocholic acid (0.3 μM) and TAS2R4 agonist colchicine (1 mM) (Figure S4) (PDF)

## References

[ref1] Bozkurt S. B.; Hakki S. S.; Hakki E. E.; Durak Y.; Kantarci A. Porphyromonas gingivalis Lipopolysaccharide Induces a Pro-inflammatory Human Gingival Fibroblast Phenotype. Inflammation 2017, 40, 144–153. 10.1007/s10753-016-0463-7. 27812843

[ref2] Brewer D. B. The fibroblast. Proc. R. Soc. Med. 1967, 60, 778–782. 10.1177/003591576706000829. 6035406 PMC1901895

[ref3] Komuro T. Re-evaluation of fibroblasts and fibroblast-like cells. Anat. Embryol. 1990, 182, 103–112. 10.1007/BF00174011. 2244684

[ref4] Vaday G. G.; Lider O. Extracellular matrix moieties, cytokines, and enzymes: dynamic effects on immune cell behavior and inflammation. J. Leukocyte Biol. 2000, 67, 149–159. 10.1002/jlb.67.2.149. 10670574

[ref5] Ehrnhöfer-Ressler M. M.; Fricke K.; Pignitter M.; Walker J. M.; Walker J.; Rychlik M.; Somoza V. Identification of 1,8-cineole, borneol, camphor, and thujone as anti-inflammatory compounds in a Salvia officinalis L. infusion using human gingival fibroblasts. J. Agric. Food Chem. 2013, 61, 3451–3459. 10.1021/jf305472t. 23488631

[ref6] Walker J. M.; Maitra A.; Walker J.; Ehrnhoefer-Ressler M. M.; Inui T.; Somoza V. Identification of *Magnolia officinalis* L. bark extract as the most potent anti-inflammatory of four plant extracts. Am. J. Chin. Med. 2013, 41, 531–544. 10.1142/S0192415X13500389. 23711140

[ref7] Josino Soares D.; Walker J.; Pignitter M.; Walker J. M.; Imboeck J. M.; Ehrnhoefer-Ressler M. M.; Montenegro Brasil I.; Somoza V. Pitanga (Eugenia uniflora L.) fruit juice and two major constituents thereof exhibit anti-inflammatory properties in human gingival and oral gum epithelial cells. Food Funct. 2014, 5, 2981–2988. 10.1039/c4fo00509k. 25228206

[ref8] Schueller K.; Hans J.; Pfeiffer S.; Walker J.; Ley J. P.; Somoza V. Identification of Interleukin-8-Reducing Lead Compounds Based on SAR Studies on Dihydrochalcone-Related Compounds in Human Gingival Fibroblasts (HGF-1 cells) In Vitro. Molecules 2020, 25, 1382 10.3390/molecules25061382. 32197426 PMC7144391

[ref9] Tiroch J.; Sterneder S.; Di Pizio A.; Lieder B.; Hoelz K.; Holik A. K.; Pignitter M.; Behrens M.; Somoza M.; Ley J. P.; Somoza V. Bitter Sensing TAS2R50 Mediates the trans-Resveratrol-Induced Anti-inflammatory Effect on Interleukin 6 Release in HGF-1 Cells in Culture. J. Agric. Food Chem. 2021, 69, 13339–13349. 10.1021/acs.jafc.0c07058. 33461297

[ref10] Zhou Z.; Xi R.; Liu J.; Peng X.; Zhao L.; Zhou X.; Li J.; Zheng X.; Xu X. TAS2R16 Activation Suppresses LPS-Induced Cytokine Expression in Human Gingival Fibroblasts. Front. Immunol. 2021, 12, 726546 10.3389/fimmu.2021.726546. 34975834 PMC8714777

[ref11] Behrens M.; Born S.; Redel U.; Voigt N.; Schuh V.; Raguse J. D.; Meyerhof W. Immunohistochemical detection of TAS2R38 protein in human taste cells. PLoS One 2012, 7, e40304 10.1371/journal.pone.0040304. 22792271 PMC3391245

[ref12] Behrens M.; Foerster S.; Staehler F.; Raguse J. D.; Meyerhof W. Gustatory expression pattern of the human TAS2R bitter receptor gene family reveals a heterogenous population of bitter responsive taste receptor cells. J. Neurosci. 2007, 27, 12630–12640. 10.1523/JNEUROSCI.1168-07.2007. 18003842 PMC6673335

[ref13] Spaggiari G.; Di Pizio A.; Cozzini P. Sweet, umami and bitter taste receptors: State of the art of in silico molecular modeling approaches. Trends Food Sci. Technol. 2020, 96, 21–29. 10.1016/j.tifs.2019.12.002.

[ref14] Frank O.; Ottinger H.; Hofmann T. Characterization of an Intense Bitter-Tasting 1H,4H-Quinolizinium-7-olate by Application of the Taste Dilution Analysis, a Novel Bioassay for the Screening and Identification of Taste-Active Compounds in Foods. J. Agric. Food Chem. 2001, 49, 231–238. 10.1021/jf0010073. 11170582

[ref15] Dagan-Wiener A.; Di Pizio A.; Nissim I.; Bahia M. S.; Dubovski N.; Margulis E.; Niv M. Y. BitterDB: taste ligands and receptors database in 2019. Nucleic Acids Res. 2019, 47, D1179–D1185. 10.1093/nar/gky974. 30357384 PMC6323989

[ref16] Luo G.; Somoza V.; Dunkel A. https://fsbi-db.de/.

[ref17] Bayer S.; Mayer A. I.; Borgonovo G.; Morini G.; Di Pizio A.; Bassoli A. Chemoinformatics View on Bitter Taste Receptor Agonists in Food. J. Agric. Food Chem. 2021, 69, 13916–13924. 10.1021/acs.jafc.1c05057. 34762411 PMC8630789

[ref18] Lossow K.; Hübner S.; Roudnitzky N.; Slack J. P.; Pollastro F.; Behrens M.; Meyerhof W. Comprehensive Analysis of Mouse Bitter Taste Receptors Reveals Different Molecular Receptive Ranges for Orthologous Receptors in Mice and Humans. J. Biol. Chem. 2016, 291, 15358–15377. 10.1074/jbc.M116.718544. 27226572 PMC4946946

[ref19] Meyerhof W.; Batram C.; Kuhn C.; Brockhoff A.; Chudoba E.; Bufe B.; Appendino G.; Behrens M. The molecular receptive ranges of human TAS2R bitter taste receptors. Chem. Senses 2010, 35, 157–170. 10.1093/chemse/bjp092. 20022913

[ref20] Behrens M.; Redel U.; Blank K.; Meyerhof W. The human bitter taste receptor TAS2R7 facilitates the detection of bitter salts. Biochem. Biophys. Res. Commun. 2019, 512, 877–881. 10.1016/j.bbrc.2019.03.139. 30928101

[ref21] Wang Y.; Zajac A. L.; Lei W.; Christensen C. M.; Margolskee R. F.; Bouysset C.; Golebiowski J.; Zhao H.; Fiorucci S.; Jiang P. Metal Ions Activate the Human Taste Receptor TAS2R7. Chem. Senses 2019, 44, 339–347. 10.1093/chemse/bjz024. 31066447 PMC6538953

[ref22] Bufe B.; Hofmann T.; Krautwurst D.; Raguse J. D.; Meyerhof W. The human TAS2R16 receptor mediates bitter taste in response to beta-glucopyranosides. Nat. Genet. 2002, 32, 397–401. 10.1038/ng1014. 12379855

[ref23] Mueller K. L.; Hoon M. A.; Erlenbach I.; Chandrashekar J.; Zuker C. S.; Ryba N. J. P. The receptors and coding logic for bitter taste. Nature 2005, 434, 225–229. 10.1038/nature03352. 15759003

[ref24] Bufe B.; Breslin P. A.; Kuhn C.; Reed D. R.; Tharp C. D.; Slack J. P.; Kim U.-K.; Drayna D.; Meyerhof W. The molecular basis of individual differences in phenylthiocarbamide and propylthiouracil bitterness perception. Curr. Biol. 2005, 15, 322–327. 10.1016/j.cub.2005.01.047. 15723792 PMC1400547

[ref25] Kim U. K.; Jorgenson E.; Coon H.; Leppert M.; Risch N.; Drayna D. Positional cloning of the human quantitative trait locus underlying taste sensitivity to phenylthiocarbamide. Science 2003, 299, 1221–1225. 10.1126/science.1080190. 12595690

[ref26] Riedel K.; Sombroek D.; Fiedler B.; Siems K.; Krohn M. Human cell-based taste perception - a bittersweet job for industry. Nat. Prod. Rep. 2017, 34, 484–495. 10.1039/c6np00123h. 28393162

[ref27] Liszt K. I.; Ley J. P.; Lieder B.; Behrens M.; Stöger V.; Reiner A.; Hochkogler C. M.; Köck E.; Marchiori A.; Hans J.; Widder S.; Krammer G.; Sanger G. J.; Somoza M. M.; Meyerhof W.; Somoza V. Caffeine induces gastric acid secretion via bitter taste signaling in gastric parietal cells. Proc. Natl. Acad. Sci. U. S. A. 2017, 114, E6260–E6269. 10.1073/pnas.1703728114. 28696284 PMC5544304

[ref28] Walker J.; Hell J.; Liszt K. I.; Dresel M.; Pignitter M.; Hofmann T.; Somoza V. Identification of beer bitter acids regulating mechanisms of gastric acid secretion. J. Agric. Food Chem. 2012, 60, 1405–1412. 10.1021/jf204306z. 22313115

[ref29] Liszt K. I.; Hans J.; Ley J. P.; Köck E.; Somoza V. Characterization of Bitter Compounds via Modulation of Proton Secretion in Human Gastric Parietal Cells in Culture. J. Agric. Food Chem. 2018, 66, 2295–2300. 10.1021/acs.jafc.7b01051. 28525714

[ref30] Zhang Y.; Yuan L.; Meng L.; Fang M.; Guo S.; Wang D.; Ma J.; Wang L. Guanine and nucleotide binding protein 3 promotes odonto/osteogenic differentiation of apical papilla stem cells via JNK and ERK signaling pathways. Int. J. Mol. Med. 2018, 43, 382–392. 10.3892/ijmm.2018.3984. 30431055 PMC6257834

[ref31] McLaughlin S. K.; McKinnon P. J.; Spickofsky N.; Danho W.; Margolskee R. F. Molecular cloning of G proteins and phosphodiesterases from rat taste cells. Physiol. Behav. 1994, 56, 1157–1164. 10.1016/0031-9384(94)90360-3. 7878085

[ref32] Dunkel A.; Hofmann T.; Di Pizio A. In Silico Investigation of Bitter Hop-Derived Compounds and Their Cognate Bitter Taste Receptors. J. Agric. Food Chem. 2020, 68, 10414–10423. 10.1021/acs.jafc.9b07863. 32027492

[ref33] Behrens M.; Brockhoff A.; Batram C.; Kuhn C.; Appendino G.; Meyerhof W. The human bitter taste receptor hTAS2R50 is activated by the two natural bitter terpenoids andrographolide and amarogentin. J. Agric. Food Chem. 2009, 57, 9860–9866. 10.1021/jf9014334. 19817411

[ref34] Ley J. P.; Krammer G.; Reinders G.; Gatfield I. L.; Bertram H. J. Evaluation of bitter masking flavanones from Herba Santa (Eriodictyon californicum (H. and A.) Torr., Hydrophyllaceae). J. Agric. Food Chem. 2005, 53, 6061–6066. 10.1021/jf0505170. 16028996

[ref35] Singldinger B.; Dunkel A.; Bahmann D.; Bahmann C.; Kadow D.; Bisping B.; Hofmann T. New Taste-Active 3-( O-beta-d-Glucosyl)-2-oxoindole-3-acetic Acids and Diarylheptanoids in Cimiciato-Infected Hazelnuts. J. Agric. Food Chem. 2018, 66, 4660–4673. 10.1021/acs.jafc.8b01216. 29649863

[ref36] Szejtli J.; Szente L. Elimination of bitter, disgusting tastes of drugs and foods by cyclodextrins. Eur. J. Pharm. Biopharm. 2005, 61, 115–125. 10.1016/j.ejpb.2005.05.006. 16185857

[ref37] Wadhwa J.; Puri S. Taste Masking: A Novel Approch For Bitter And Obnoxious Drugs. Int. J. Pharm. Prof.. Res 2011, 11, 1–11.

[ref38] Intelmann D.; Batram C.; Kuhn C.; Haseleu G.; Meyerhof W.; Hofmann T. Three TAS2R Bitter Taste Receptors Mediate the Psychophysical Responses to Bitter Compounds of Hops (Humulus lupulus L.) and Beer. Chemosens. Percept. 2009, 2, 118–132. 10.1007/s12078-009-9049-1.

[ref39] Glabasnia A.; Dunkel A.; Frank O.; Hofmann T. Decoding the Nonvolatile Sensometabolome of Orange Juice ( Citrus sinensis). J. Agric. Food Chem. 2018, 66, 2354–2369. 10.1021/acs.jafc.7b06142. 29430918

[ref40] Stark T.; Bareuther S.; Hofmann T. Molecular definition of the taste of roasted cocoa nibs (Theobroma cacao) by means of quantitative studies and sensory experiments. J. Agric. Food Chem. 2006, 54, 5530–5539. 10.1021/jf0608726. 16848542

[ref41] Intelmann D.; Kummerlöwe G.; Haseleu G.; Desmer N.; Schulze K.; Fröhlich R.; Frank O.; Luy B.; Hofmann T. Structures of storage-induced transformation products of the beer’s bitter principles, revealed by sophisticated NMR spectroscopic and LC-MS techniques. Chemistry 2009, 15, 13047–13058. 10.1002/chem.200902058. 19876978

[ref42] Frank O.; Kreissl J. K.; Daschner A.; Hofmann T. Accurate determination of reference materials and natural isolates by means of quantitative (1)h NMR spectroscopy. J. Agric. Food Chem. 2014, 62, 2506–2515. 10.1021/jf405529b. 24559241

[ref43] Glabasnia A.; Hofmann T. Sensory-directed identification of taste-active ellagitannins in American (Quercus alba L.) and European oak wood (Quercus robur L.) and quantitative analysis in bourbon whiskey and oak-matured red wines. J. Agric. Food Chem. 2006, 54, 3380–3390. 10.1021/jf052617b. 16637699

[ref44] Hasegawa S.; Berhow M. A.; Fong C. H., Analysis of Bitter Principles in Citrus. In Fruit Analysis; Linskens H. F.; Jackson J. F., Eds. Springer Berlin Heidelberg: Berlin, Heidelberg, 1996; pp. 59–80.

[ref45] Keast R. S. J.; Roper J. A complex relationship among chemical concentration, detection threshold, and suprathreshold intensity of bitter compounds. Chem. Senses 2007, 32, 245–253. 10.1093/chemse/bjl052. 17220518

[ref46] Stoeger V.; Liszt K. I.; Lieder B.; Wendelin M.; Zopun M.; Hans J.; Ley J. P.; Krammer G. E.; Somoza V. Identification of Bitter-Taste Intensity and Molecular Weight as Amino Acid Determinants for the Stimulating Mechanisms of Gastric Acid Secretion in Human Parietal Cells in Culture. J. Agric. Food Chem. 2018, 66, 6762–6771. 10.1021/acs.jafc.8b01802. 29879844

[ref47] Gillespie M.; Jassal B.; Stephan R.; Milacic M.; Rothfels K.; Senff-Ribeiro A.; Griss J.; Sevilla C.; Matthews L.; Gong C.; Deng C.; Varusai T.; Ragueneau E.; Haider Y.; May B.; Shamovsky V.; Weiser J.; Brunson T.; Sanati N.; Beckman L.; Shao X.; Fabregat A.; Sidiropoulos K.; Murillo J.; Viteri G.; Cook J.; Shorser S.; Bader G.; Demir E.; Sander C.; Haw R.; Wu G.; Stein L.; Hermjakob H.; D’Eustachio P. The reactome pathway knowledgebase 2022. Nucleic Acids Res. 2022, 50, D687–D692. 10.1093/nar/gkab1028. 34788843 PMC8689983

[ref48] Siegel S.; Castellan N. J., Non Parametric Statistics for the Behavioural Sciences; MacGraw Hill Int.: New York: 1988.

[ref49] Narukawa M.; Noga C.; Ueno Y.; Sato T.; Misaka T.; Watanabe T. Evaluation of the bitterness of green tea catechins by a cell-based assay with the human bitter taste receptor hTAS2R39. Biochem. Biophys. Res. Commun. 2011, 405, 620–625. 10.1016/j.bbrc.2011.01.079. 21272567

[ref50] de Graaf C.; Foata N.; Engkvist O.; Rognan D. Molecular modeling of the second extracellular loop of G-protein coupled receptors and its implication on structure-based virtual screening. Proteins 2008, 71, 599–620. 10.1002/prot.21724. 17972285

[ref51] Born S.; Levit A.; Niv M. Y.; Meyerhof W.; Behrens M. The human bitter taste receptor TAS2R10 is tailored to accommodate numerous diverse ligands. J. Neurosci. 2013, 33, 201–213. 10.1523/JNEUROSCI.3248-12.2013. 23283334 PMC6618634

[ref52] Jaiteh M.; Rodríguez-Espigares I.; Selent J.; Carlsson J. Performance of virtual screening against GPCR homology models: Impact of template selection and treatment of binding site plasticity. PLoS Comput. Biol. 2020, 16, e1007680 10.1371/journal.pcbi.1007680. 32168319 PMC7135368

[ref53] Nicoli A.; Dunkel A.; Giorgino T.; de Graaf C.; Di Pizio A. Classification Model for the Second Extracellular Loop of Class A GPCRs. J. Chem. Inf. Model. 2022, 62, 511–522. 10.1021/acs.jcim.1c01056. 35113559

[ref54] Reichling C.; Meyerhof W.; Behrens M. Functions of human bitter taste receptors depend on N-glycosylation. J. Neurochem. 2008, 106, 1138–1148. 10.1111/j.1471-4159.2008.05453.x. 18466324

[ref55] Meyerhof W. Elucidation of mammalian bitter taste. Rev. Physiol., Biochem. Pharmacol. 2005, 154, 37–72. 10.1007/s10254-005-0041-0. 16032395

[ref56] Behrens M.; Brockhoff A.; Kuhn C.; Bufe B.; Winnig M.; Meyerhof W. The human taste receptor hTAS2R14 responds to a variety of different bitter compounds. Biochem. Biophys. Res. Commun. 2004, 319, 479–485. 10.1016/j.bbrc.2004.05.019. 15178431

[ref57] Workman A. D.; Palmer J. N.; Adappa N. D.; Cohen N. A. The Role of Bitter and Sweet Taste Receptors in Upper Airway Immunity. Curr. Allergy Asthma Rep. 2015, 15, 72 10.1007/s11882-015-0571-8. 26492878 PMC4830640

[ref58] Patel N. N.; Workman A. D.; Cohen N. A. Role of Taste Receptors as Sentinels of Innate Immunity in the Upper Airway. J. Pathog. 2018, 2018, 9541987 10.1155/2018/9541987. 30363975 PMC6188595

[ref59] Deshpande D. A.; Wang W. C. H.; McIlmoyle E. L.; Robinett K. S.; Schillinger R. M.; An S. S.; Sham J. S.; Liggett S. B. Bitter taste receptors on airway smooth muscle bronchodilate by localized calcium signaling and reverse obstruction. Nat. Med. 2010, 16, 1299–1304. 10.1038/nm.2237. 20972434 PMC3066567

[ref60] Wu S. V.; Rozengurt N.; Yang M.; Young S. H.; Sinnett-Smith J.; Rozengurt E. Expression of bitter taste receptors of the T2R family in the gastrointestinal tract and enteroendocrine STC-1 cells. Proc. Natl. Acad. Sci. U. S. A. 2002, 99, 2392–2397. 10.1073/pnas.042617699. 11854532 PMC122375

[ref61] Singh N.; Vrontakis M.; Parkinson F.; Chelikani P. Functional bitter taste receptors are expressed in brain cells. Biochem. Biophys. Res. Commun. 2011, 406, 146–151. 10.1016/j.bbrc.2011.02.016. 21303656

[ref62] Deckmann K.; Filipski K.; Krasteva-Christ G.; Fronius M.; Althaus M.; Rafiq A.; Papadakis T.; Renno L.; Jurastow I.; Wessels L.; Wolff M.; Schütz B.; Weihe E.; Chubanov V.; Gudermann T.; Klein J.; Bschleipfer T.; Kummer W. Bitter triggers acetylcholine release from polymodal urethral chemosensory cells and bladder reflexes. Proc. Natl. Acad. Sci. U. S. A. 2014, 111, 8287–8292. 10.1073/pnas.1402436111. 24843119 PMC4050540

[ref63] Xu J.; Cao J.; Iguchi N.; Riethmacher D.; Huang L. Functional characterization of bitter-taste receptors expressed in mammalian testis. Mol. Hum. Reprod. 2013, 19, 17–28. 10.1093/molehr/gas040. 22983952 PMC3521485

[ref64] Malki A.; Fiedler J.; Fricke K.; Ballweg I.; Pfaffl M. W.; Krautwurst D. Class I odorant receptors, TAS1R and TAS2R taste receptors, are markers for subpopulations of circulating leukocytes. J. Leukocyte Biol. 2015, 97, 533–545. 10.1189/jlb.2A0714-331RR. 25624459 PMC5477889

[ref65] Li Z. W.; Sun B.; Gong T.; Guo S.; Zhang J.; Wang J.; Sugawara A.; Jiang M.; Yan J.; Gurary A.; Zheng X.; Gao B.; Xiao S. Y.; Chen W.; Ma C.; Farrar C.; Zhu C.; Chan O. T. M.; Xin C.; Winnicki A.; Winnicki J.; Tang M.; Park R.; Winnicki M.; Diener K.; Wang Z.; Liu Q.; Chu C. H.; Arter Z. L.; Yue P.; Alpert L.; Hui G. S.; Fei P.; Turkson J.; Yang W.; Wu G.; Tao A.; Ramos J. W.; Moisyadi S.; Holcombe R. F.; Jia W.; Birnbaumer L.; Zhou X.; Chu W. M. GNAI1 and GNAI3 Reduce Colitis-Associated Tumorigenesis in Mice by Blocking IL6 Signaling and Down-regulating Expression of GNAI2. Gastroenterology 2019, 156, 2297–2312. 10.1053/j.gastro.2019.02.040. 30836096 PMC6628260

[ref66] Luo X. C.; Chen Z. H.; Xue J. B.; Zhao D. X.; Lu C.; Li Y. H.; Li S. M.; Du Y. W.; Liu Q.; Wang P.; Liu M.; Huang L. Infection by the parasitic helminth Trichinella spiralis activates a Tas2r-mediated signaling pathway in intestinal tuft cells. Proc. Natl. Acad. Sci. U. S. A. 2019, 116, 5564–5569. 10.1073/pnas.1812901116. 30819885 PMC6431192

[ref67] Lee R. J.; Chen B.; Redding K. M.; Margolskee R. F.; Cohen N. A. Mouse nasal epithelial innate immune responses to Pseudomonas aeruginosa quorum-sensing molecules require taste signaling components. Innate Immun. 2014, 20, 606–617. 10.1177/1753425913503386. 24045336 PMC4811369

[ref68] Grassin-Delyle S.; Salvator H.; Mantov N.; Abrial C.; Brollo M.; Faisy C.; Naline E.; Couderc L. J.; Devillier P. Bitter Taste Receptors (TAS2Rs) in Human Lung Macrophages: Receptor Expression and Inhibitory Effects of TAS2R Agonists. Front. Physiol. 2019, 10, 1267 10.3389/fphys.2019.01267. 31632299 PMC6783802

[ref69] Soares S.; Silva M. S.; Garcia-Estevez I.; Groβmann P.; Brás N.; Brandão E.; Mateus N.; de Freitas V.; Behrens M.; Meyerhof W. Human Bitter Taste Receptors Are Activated by Different Classes of Polyphenols. J. Agric. Food Chem. 2018, 66, 8814–8823. 10.1021/acs.jafc.8b03569. 30056706

[ref70] Roland W. S. U.; van Buren L.; Gruppen H.; Driesse M.; Gouka R. J.; Smit G.; Vincken J.-P. Bitter taste receptor activation by flavonoids and isoflavonoids: modeled structural requirements for activation of hTAS2R14 and hTAS2R39. J. Agric. Food Chem. 2013, 61, 10454–10466. 10.1021/jf403387p. 24117141

[ref71] Verkman A. S. The quenching of an intramembrane fluorescent probe. A method to study the binding and permeation of phloretin through bilayers. Biochim. Biophys. Acta, Biomembr. 1980, 599, 370–379. 10.1016/0005-2736(80)90184-4. 7407100

[ref72] Carmosino M.; Procino G.; Casavola V.; Svelto M.; Valenti G. The cultured human gastric cells HGT-1 express the principal transporters involved in acid secretion. Pfluegers Arch. 2000, 440, 871–880. 10.1007/s004240000363. 11041553

[ref73] Beltrán L. R.; Sterneder S.; Hasural A.; Paetz S.; Hans J.; Ley J. P.; Somoza V. Reducing the Bitter Taste of Pharmaceuticals Using Cell-Based Identification of Bitter-Masking Compounds. Pharmaceuticals (Basel) 2022, 15, 317 10.3390/ph15030317. 35337115 PMC8953435

[ref74] Chandrashekar J.; Mueller K. L.; Hoon M. A.; Adler E.; Feng L.; Guo W.; Zuker C. S.; Ryba N. J. P. T2Rs Function as Bitter Taste Receptors. Cell 2000, 100, 703–711. 10.1016/S0092-8674(00)80706-0. 10761935

[ref75] Offermanns S.; Simon M. I. Gα15 and Gα16 Couple a Wide Variety of Receptors to Phospholipase C. J. Biol. Chem. 1995, 270, 15175–15180. 10.1074/jbc.270.25.15175. 7797501

[ref76] Kohl S.; Behrens M.; Dunkel A.; Hofmann T.; Meyerhof W. Amino acids and peptides activate at least five members of the human bitter taste receptor family. J. Agric. Food Chem. 2013, 61, 53–60. 10.1021/jf303146h. 23214402

[ref77] Slack J. P.; Brockhoff A.; Batram C.; Menzel S.; Sonnabend C.; Born S.; Galindo M. M.; Kohl S.; Thalmann S.; Ostopovici-Halip L.; Simons C. T.; Ungureanu I.; Duineveld K.; Bologa C. G.; Behrens M.; Furrer S.; Oprea T. I.; Meyerhof W. Modulation of bitter taste perception by a small molecule hTAS2R antagonist. Curr. Biol. 2010, 20, 1104–1109. 10.1016/j.cub.2010.04.043. 20537538 PMC2925244

[ref78] Stand., I. Technical Committee ISO/TC 34. ISO 13301:2018, Sensory Analysis — Methodology — General Guidance for Measuring Odour , Flavour and Taste Detection; Elsevier Inc. 2018 .

[ref79] Dragoş D.; Petran M.; Gradinaru T. C.; Gilca M. Phytochemicals and Inflammation: Is Bitter Better?. Plants 2022, 11, 2991 10.3390/plants11212991. 36365444 PMC9654259

