# Supplementary material for: Human Gingival Fibroblasts as a Novel Cell Model Describing the Association between Bitter Taste Thresholds and Interleukin-6 Release
Source: J Agric Food Chem. 2023 Mar 21;71(13):5314–25. doi: 10.1021/acs.jafc.2c06979 (PMC10080686; doi:10.1021/acs.jafc.2c06979)

Supplemental material to

Human Gingival Fibroblasts as a Novel Cell Model Describing the Association between Bitter Taste Thresholds and Interleukin-6 Release

Johanna Tiroch[1,2], Andreas Dunkel[3], Sonja Sterneder[1,2,] Sofie Zehentner[1,2], Maik Behrens[3], Antonella Di Pizio[3], Jakob P. Ley[4], Barbara Lieder[1], Veronika Somoza[1,3,5]

Tables

Table S1.

Calculated molecular descriptors of the selected compounds: molecular weight (MW), lipophilic parameter (AlogP) and hydrogen bond acceptor/donor (HBA and HBD).

Compound	MW [g/mol]	AlogP	HBA	HBD
EGCG	458.38	2.89	11	8
IsoSNT	372.38	3.79	7	0
NAR	580.55	-0.47	14	8
QHCl	324.43	2.73	3	1
RSV	228.25	3.01	3	3
SNT	372.38	3.79	7	0

Table S2.

Results of HGT-1 and HGF-1 cell viability by means of MTT shown in percent.

	Cell viability [%] of	
	HGF-1	HGT-1
CAB 500 µM	105.3 ± 7.6	102.9 ± 10.4
DIOS 1 µM	96.0 ± 5.5	100.9 ± 9.3
EGCG 100 µM	100.5 ± 5.4	115.9 ± 4.0 (1mM)
IsoA 35.7 ppm	100.1 ± 5.7	94.2 ± 5.5
IsoSNT 25 µM	101.3 ± 6.2	100.7 ± 6.00 (0.1 µM)
L – Arg 50000 µM	99.1 ± 4.6	Stöger et al.,2018[1]
NAR 500 µM	100.7 ± 5.9	Liszt et al., 2017[2]
QHCl 40 µM	100.5 ± 10.1	106.2 ± 6.0
RSV 250 µM	103.1 ± 10.5	100 µM Tiroch et al.,2021[3]
SNT 50 µM	102.3 ± 10.2	97.2 ± 6.8
THEO 250 µM	101.0 ± 6.1	Liszt et al., 2017[2]
MAT 50µM	100.2 ± 6.1	Liszt et al., 2017[2]
HED 50 µM	100.6 ± 3.1	Liszt et al., 2017[2]

[a] Data are indicated as mean ± SD and calculated as treatment over control (non - treated cells, set to 100 %). Calculations were made from n = 3-4 and n t.r.=3. HGF-1 cells were co-incubated with *Pg*-LPS [10 µg/mL] for 6 hr. Statistics: One way ANOVA, no significant differences compared to non – treated cells, calculation for each cell line respectively.

Figure captions

Figure S1.

(A) Pathway network of TAS2R50, and IL-6 pathway by reactom. (B) Immunofluorescence analysis of GNAI3 in HGF-1 cells. Cells were stained with: DAPI (blue) as counter stain, ActinRed (red), primary 1:200 (# PA5-27940, Invivogen) or the control (lower part) without and stained with 1:200 Alexa Fluor® 488 tag (ab150081, abcam) (green).

Figure S2.

Dose-response experiments of SNT – sinensetin (0.1 – 50 µM), NAR – naringin (0.1-500 µM, EGCG – epigallocatechin gallate (0.1 – 100 µM), QHCl - quinine HCl (0.1 – 40 µM). Data are shown as raw data points from individual passages connected by black lines, overall fit coloured line: n=3 - 4 t.r. = 2.

Figure S3.

3D representation of the docking poses of RSV, NAR, SNT, EGCG and QHCl (shown as green sticks) into the TAS2R50 binding pocket. Ligand-interacting residues are shown as grey sticks, hydrogen bonds are shown as purple dashed lines, and the π−π stacking with blue lines. Docking and MM-GBSA scores of RSV, NAR, SNT, EGCG & QHCl in complex with TAS2R50, and predicted ligand interacting residues.

Figure S4.

Antagonistic effect of 10 µM MAT in TAS2R4 and TAS2R43 transfected HEK – 293T-Gα16gust44 cells by co-administration with TAS2R43 agonist aristocholic acid (0.3 µM) and TAS2R4 agonist colchicine (1 mM). Activations caused by agonists alone were set to 100%. Statistics: T-test: * $p < 0.05$, ** $p < 0.01$;n = 2, t.r. = 2

Figure S1.

A

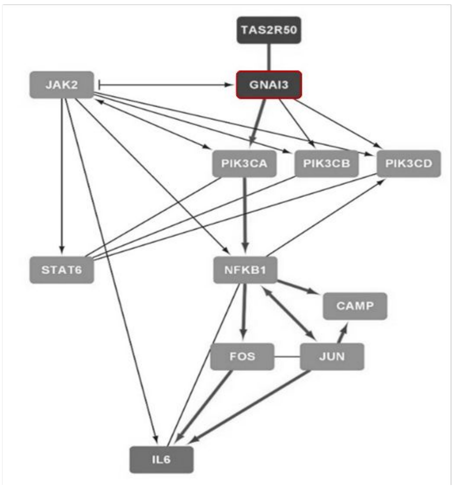
TAS2R50
JAK2
GNAI3
PIK3CA
PIK3CB
PIK3CD
STAT6
NFKB1
CAMP
FOS
JUN
IL6

B

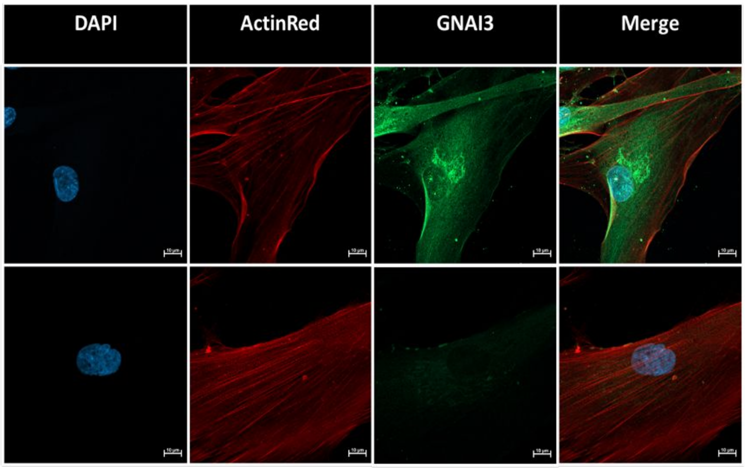
DAPI
ActinRed
GNAI3
Merge

Figure S 2.

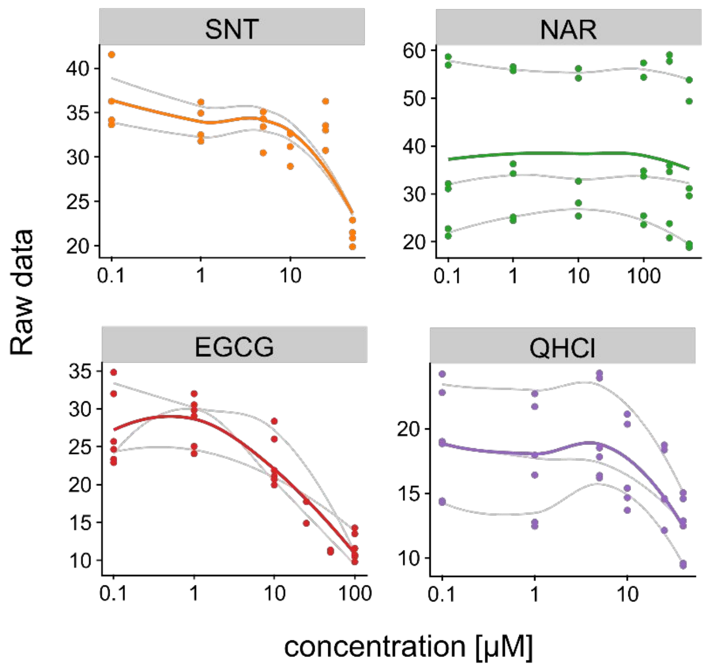

SNT
NAR
EGCG
QHCl
Raw data
40
35
30
25
20
0.1
1
10
60
50
40
30
20
0.1
1
10
100
35
30
25
20
15
10
0.1
1
10
100
20
15
10
0.1
1
10
concentration [µM]

Figure S 3.

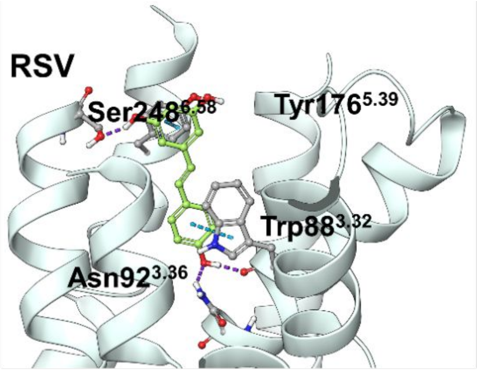

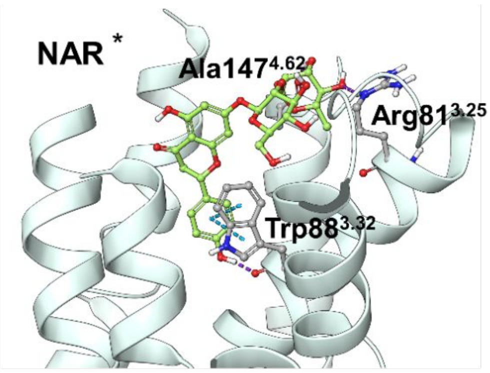

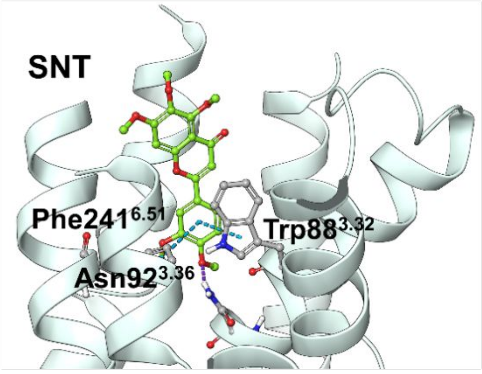

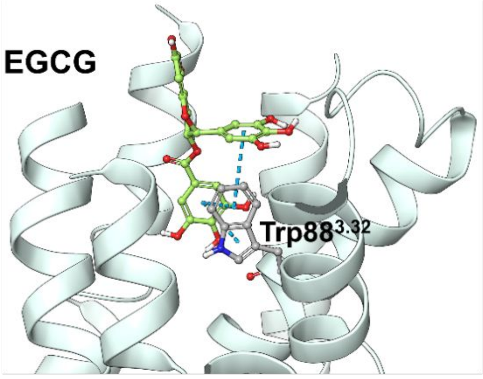

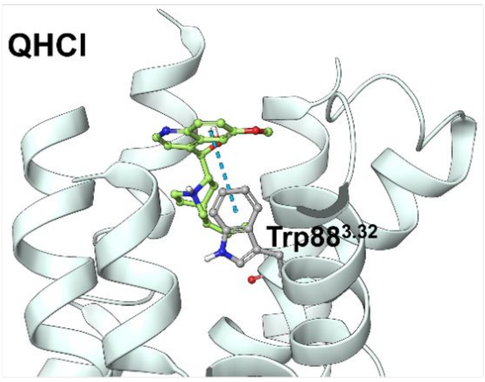

Ligand	Glide score [kcal/mol]	MM-GBSA dG Bind [kcal/mol]
RSV	-8.83	-72.21
NAR	-8.15	-89.63
SNT	-7.54	-75.03
EGCG	-5.15	-37.67
QHCl	-3.00	7.58

* At the core of the binding site, NAR could establish interactions similar to those observed for SNT and RVS. However, because of its size, NAR binding could be affected by the ECL2, though, we cannot evaluate this possibility with our model.

Figure S4.

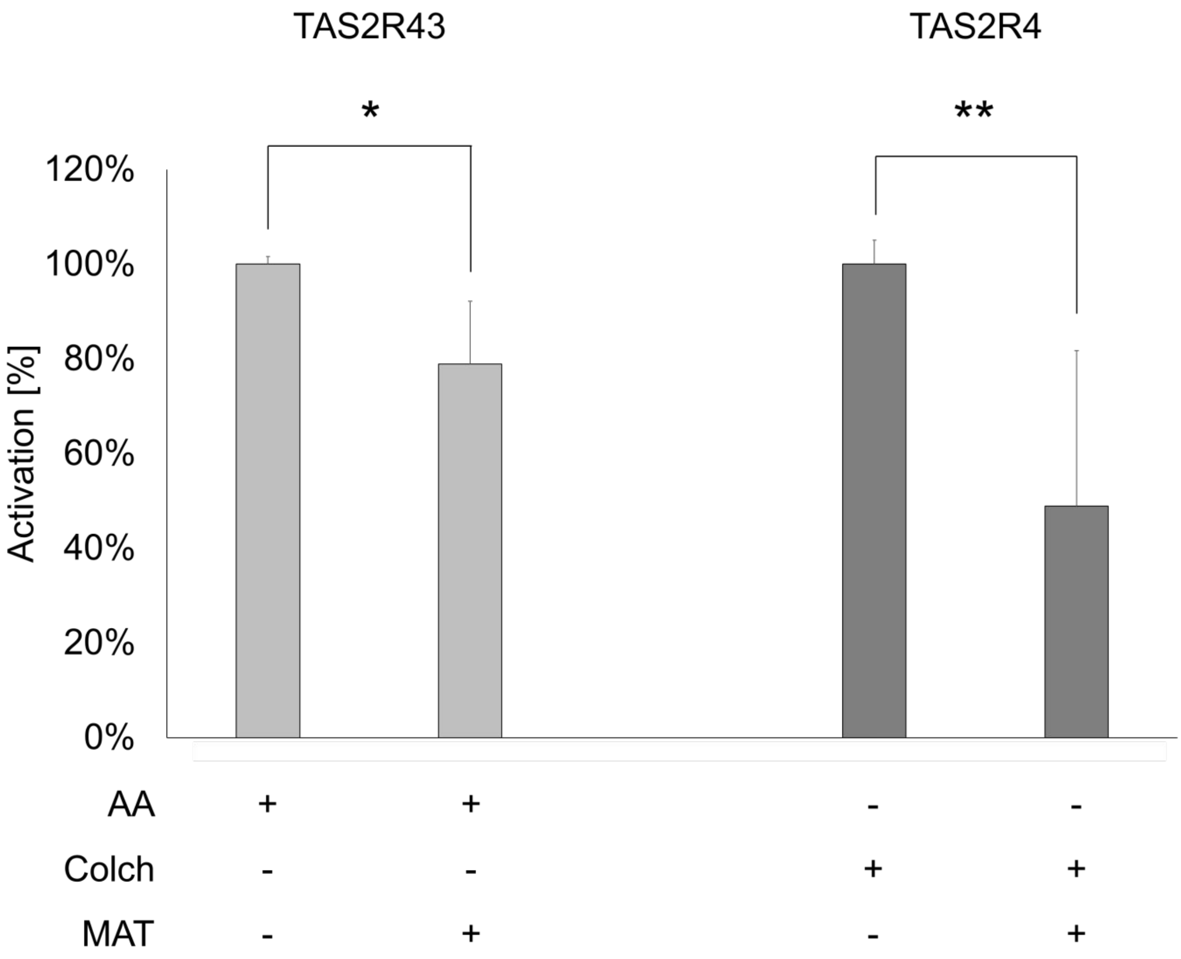

Supplement: Supplementary file 1 — jf2c06979_si_001.pdf [file jf2c06979_si_001.pdf]
